# The Fab portion of immunoglobulin G contributes to its binding to Fcγ receptor III

**DOI:** 10.1038/s41598-019-48323-w

**Published:** 2019-08-16

**Authors:** Rina Yogo, Yuki Yamaguchi, Hiroki Watanabe, Hirokazu Yagi, Tadashi Satoh, Mahito Nakanishi, Masayoshi Onitsuka, Takeshi Omasa, Mari Shimada, Takahiro Maruno, Tetsuo Torisu, Shio Watanabe, Daisuke Higo, Takayuki Uchihashi, Saeko Yanaka, Susumu Uchiyama, Koichi Kato

**Affiliations:** 1 Exploratory Research Center on Life and Living Systems (ExCELLS), National Institutes of Natural Sciences, 5-1 Higashiyama, Myodaiji, Okazaki 444-8787 Japan; 2 Graduate School of Pharmaceutical Sciences, Nagoya City University, 3-1 Tanabe-dori, Mizuho-ku, Nagoya, Aichi 467-8603 Japan; 3 Graduate School of Engineering, Osaka University, 2-1 Yamadaoka, Suita, Osaka 565-0871 Japan; 4 Biotechnology Research Institute for Drug Discovery, National Institute of Advanced Industrial Science and Technology (AIST), 1-1-1 Higashi, Central 5, Tsukuba, Ibaraki 305-8565 Japan; 5 Graduate School of Technology, Industrial and Social Sciences, Tokushima University, Minamijosanjima-cho 2-1, Tokushima, 770-8513 Japan; 6 Thermo Fisher Scientific, 3-9 Moriya-cho, Kanagawa-ku, Yokohama-shi, Kanagawa 221-0022 Japan; 7 Department of Physics, Nagoya University, Furo-cho, Chikusa-ku, Nagoya, Aichi 464-8602 Japan

**Keywords:** Molecular biophysics, Atomic force microscopy

## Abstract

Most cells active in the immune system express receptors for antibodies which mediate a variety of defensive mechanisms. These receptors interact with the Fc portion of the antibody and are therefore collectively called Fc receptors. Here, using high-speed atomic force microscopy, we observe interactions of human, humanized, and mouse/human-chimeric immunoglobulin G1 (IgG1) antibodies and their cognate Fc receptor, FcγRIIIa. Our results demonstrate that not only Fc but also Fab positively contributes to the interaction with the receptor. Furthermore, hydrogen/deuterium exchange mass spectrometric analysis reveals that the Fab portion of IgG1 is directly involved in its interaction with FcγRIIIa, in addition to the canonical Fc-mediated interaction. By targeting the previously unidentified receptor-interaction sites in IgG-Fab, our findings could inspire therapeutic antibody engineering.

## Introduction

The combination of antigen recognition and expression of effector functions typified by antibody-dependent cellular cytotoxicity (ADCC) and complement-dependent cytotoxicity is a major function of the antibody^1–4^. To exert this hub function, the antibody structure is divided into Fab arms and Fc stem. The Fab portions exhibit sequence variability in the N-terminal domains V_H_ and V_L_, which recognize various antigens, followed by constant domains C_L_ and C_H_1. By contrast, the Fc region has a two-fold-symmetric homodimeric structure comprising two C_H_2 and two C_H_3 domains. The relative orientation of the Fab arms with respect to the Fc stem varies because of their connection through a flexible linker called a hinge^1,5^.

The Fc portion of immunoglobulin G (IgG) provides interaction sites for effector molecules such as complement^1–4^. Most of the cells working in the immune system express receptors for IgG, possessing extracellular regions comprising Ig-fold domains and interacting with the Fc portion of IgG^6–11^. Therefore, they are collectively termed Fcγ receptors (FcγRs). FcγRs are classified into three major isoforms: FcγRI, FcγRII, and FcγRIII, each exhibiting different binding affinities to the IgG isotypes, and distinct expression profiles on immune cells^12–14^. Human FcγRIII is further divided into two isoforms—transmembrane FcγRIIIa and glycosylphosphatidylinositol-linked FcγRIIIb—that share 96% amino acid sequence identity in their extracellular regions. FcγRIIIa, expressed primarily on natural killer cells, promotes ADCC by interacting with the IgG in complex with antigen, whereas FcγRIIIb, expressed exclusively on neutrophils, mediates the degranulation and phagocytosis of the IgG-labeled target cells^15–19^.

The interaction modes of human IgG1 and FcγRIII molecules have been structurally characterized by X-ray crystallography using the Fc fragments and the soluble forms of FcγRIII (sFcγRIII) molecules, comprising the D1 and D2 domains^20–25^. These studies have identified their primary interaction sites—namely, the hinge-proximal segments in the C_H_2 domains of Fc and the loops in the membrane-proximal D2 domain in FcγRs—and also revealed the domain rearrangements in both proteins. Furthermore, the functional significance of intermolecular carbohydrate-carbohydrate interactions has been underscored in the interaction between human IgG1-Fc and the extracellular region of FcγRIIIa, considered to be one of the most critical factors for clinical applications of human IgG1-based therapeutic antibodies that target cancers^22,23,26–28^. However, a large gulf exists between the structural views thus obtained and the functional and therapeutic insights gained from observations under physiologically realistic conditions where IgG1 interacts with FcγRIIIa anchored on the cell surfaces through a C-terminal transmembrane segment. Here, using high-speed atomic force microscopy (HS-AFM) and human, humanized, and mouse/human-chimeric IgG1 antibodies (Supplementary Fig. 1) and their Fc fragments, along with human sFcγRIIIa immobilized through its C-terminal segment on the scanning surface, we perform real-time observation of IgG-FcγR interactions. The interaction modes of these IgGs with sFcγRIIIa have also been characterized by hydrogen-deuterium exchange mass spectrometry (HDX-MS) in solution.

## Results

### HS-AFM observations of IgG1-FcγRIIIa

**Figure 1:**
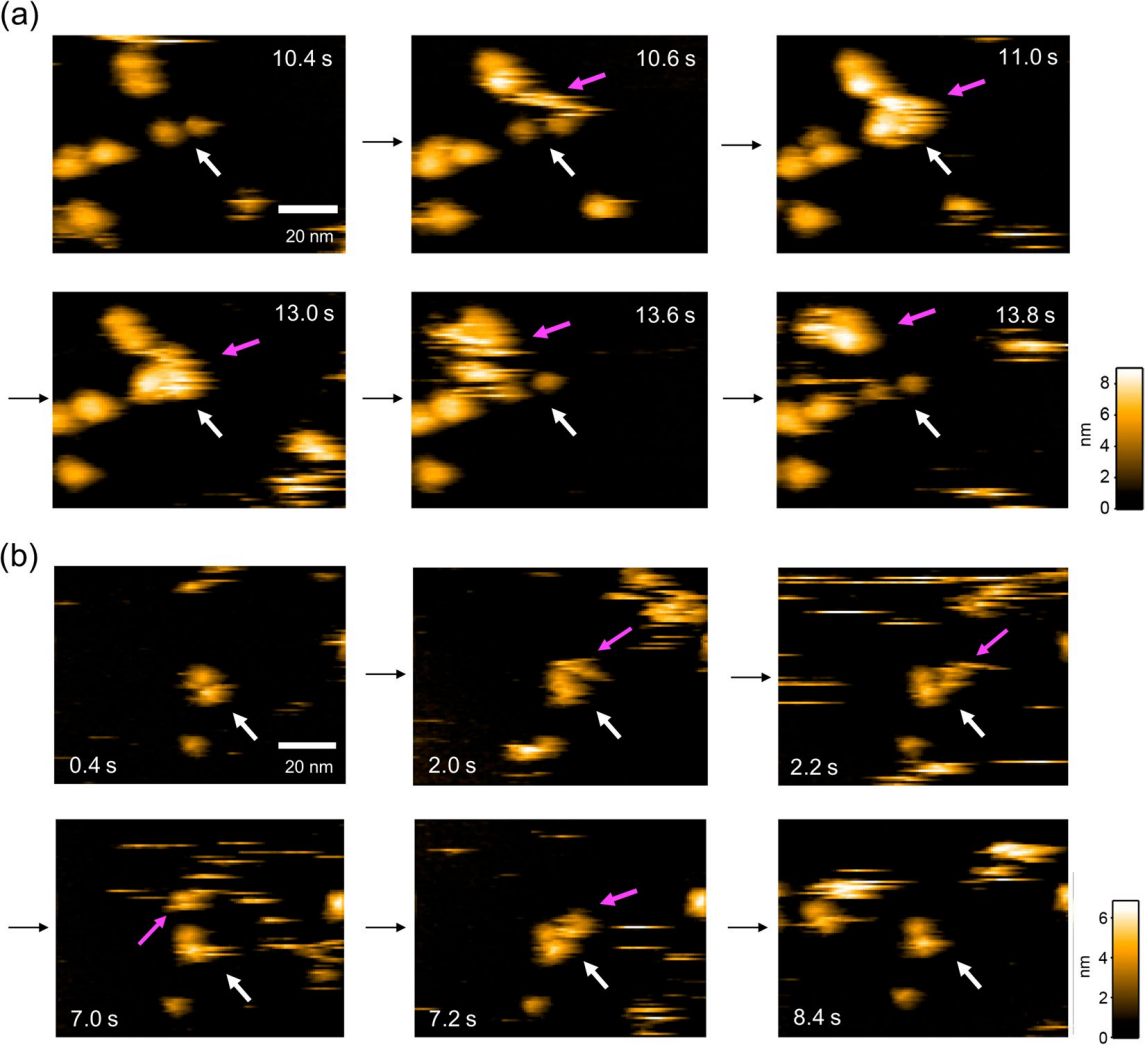
HS-AFM observations of interactions between IgG1 antibodies and their Fc fragments with sFcγRIIIa. (**a**) We indicate full-length rituximab by a red arrow (Supplementary Videos 1–3). (**b**) We indicate rituximab Fc by a magenta arrow (Supplementary Videos 4–6). In (**a**,**b**), we show an immobilized sFcγRIIIa molecule having two extracellular domains by a white arrow.

**Figure 2:**
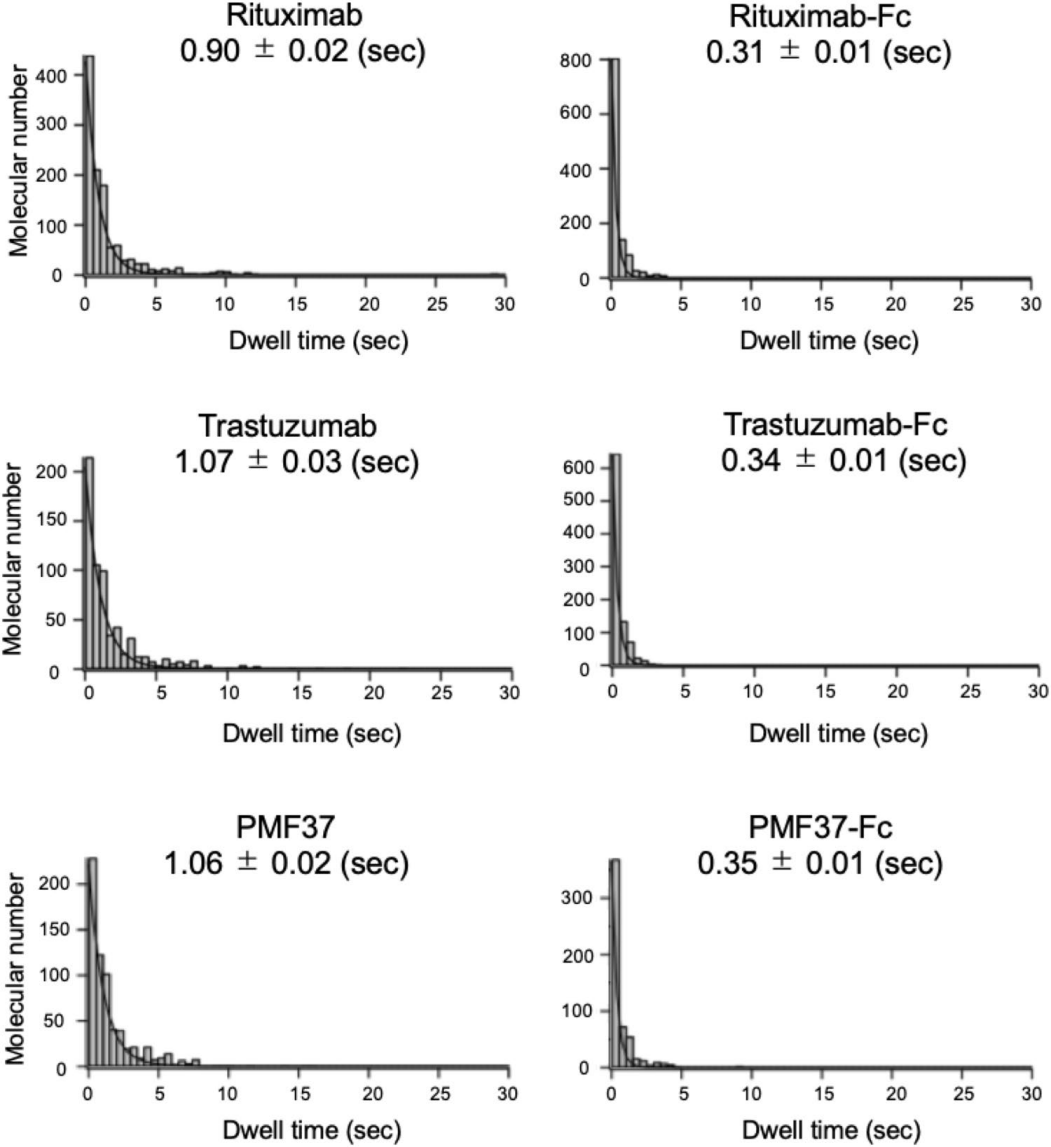
Dwell times of three kinds of IgG1 and their Fc fragments on sFcγRIIIa.

We prepared a recombinant sFcγRIIIa glycoprotein with a C-terminal hexahistidine moiety for immobilization onto a Ni^2+^-coated mica surface. When the IgG solution was added to the observation buffer in the sample chamber of HS-AFM, IgG molecules visited the extracellular domains of FcγRIIIa, transiently forming a 1:1 complex (Supplementary Videos 1–3). We compared interactions of a panel of IgGs by HS-AFM imaging (Fig. 1a) by quantifying dwell times of the IgG molecules and summarize them in Fig. 2. The human, humanized, and mouse/chimeric IgG1 antibodies—PMF37, trastuzumab, and rituximab, respectively—exhibited comparable dwell times of around 1 s.

We subjected the Fc fragments derived from these IgGs to HS-AFM observation (Fig. 1b, Supplementary Videos 4–6). Unexpectedly, dwell times of the Fc fragments were remarkably decreased (0.31 s–0.35 s) in comparison with those of the intact IgGs (Fig. 2). We confirmed that mock-treated control (PMF37 incubated in the absence of papain) maintained the longer dwell time (Supplementary Fig. 2). These data indicate that the Fab portions of IgG contribute positively to its interaction with FcγRIIIa.

### HDX-MS characterization of IgG1-FcγRIIIa interaction

**Figure 3:**
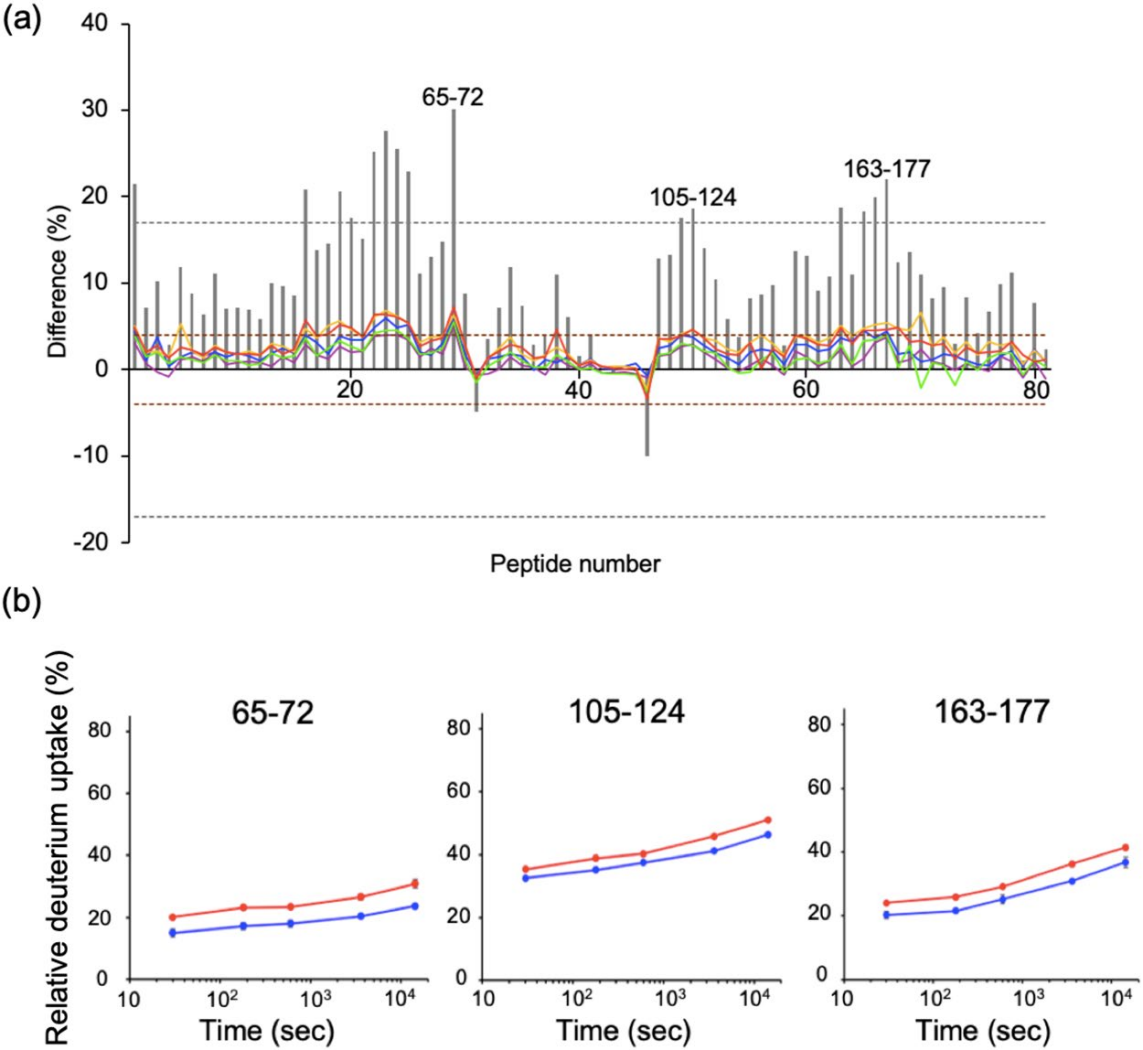
HDX-MS analysis of the light chain of PMF37. (**a**) Differential plots of deuterium uptake degrees of peptides, showing time courses; 30 s (purple), 180 s (blue), 600 s (green), 3,600 s (yellow), and 14,400 s (red), along with their summational results (gray bar). We considered that amide proton environments were significantly different between free and bound states, with 99% confidence by independent t-test, when the summational result of a corresponding peptide showed difference more than 17% (gray dashed lines) and the result at a specific exposure time of the peptide showed difference more than 4% (brown dashed lines). (**b**) Deuterium uptake curves for the representative peptides showing significant differences between free (red) and bound (blue) states.

**Figure 4:**
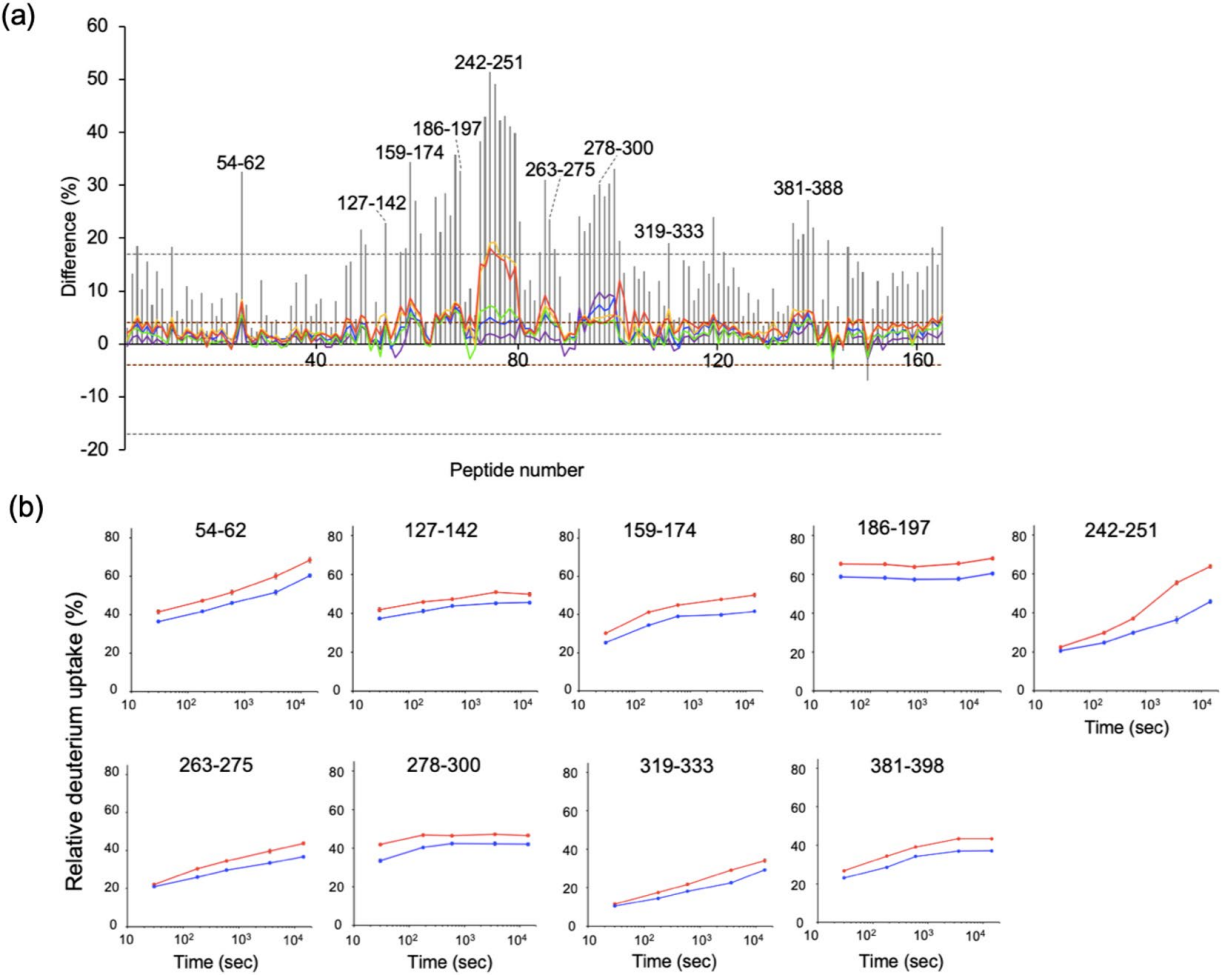
HDX-MS analysis of the heavy chain of PMF37. (**a**) Differential plots of deuterium uptake degrees of peptides, showing time courses; 30 s (purple), 180 s (blue), 600 s (green), 3,600 s (yellow), and 14,400 s (red), along with their summational results (gray bar). The criterion of significance of deuterium uptake difference between free and bound states is the same as that in Fig. 3. (**b**) Deuterium uptake curves for the representative peptides showing significant differences between free (red) and bound (blue) states.

**Figure 5:**
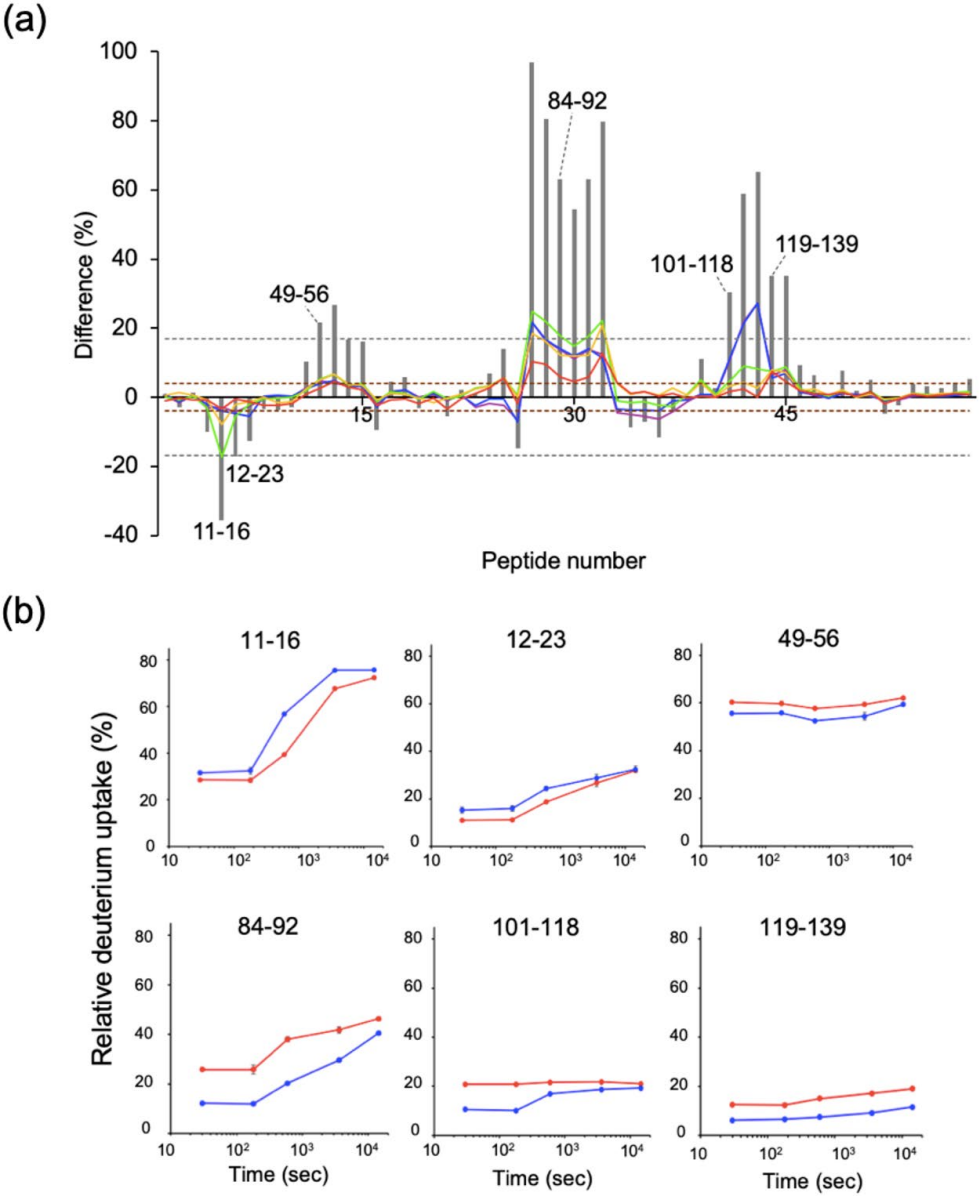
HDX-MS analysis of sFcγRIIIa. (**a**) Differential plots of deuterium uptake degrees of peptides, showing time courses; 30 s (purple), 60 s (blue), 600 s (green), 3,600 s (yellow), and 14,400 s (red), along with their summational results (gray bar). The criterion of significance of deuterium uptake difference between free and bound states is the same as that in Fig. 3. (**b**) Deuterium uptake curves for the representative peptides showing significant differences between free (red) and bound (blue) states.

The HS-AFM observation raised the possibility of direct involvement of the Fab portion of IgG1 in the interaction with sFcγRIIIa. Hence, using the human IgG1 PMF37, we attempted to identify the interaction sites of IgG1 and sFcγRIIIa by HDX-MS analysis. In this experiment, at least over 80% coverage was achieved for PMF37 and sFcγRIIIa glycoproteins, thereby enabling HDX-based detection of changes in solvent exposure of their peptide segments upon their interactions. We subjected the HDX-MS results from three independent experiments to t-test with 99% confidence and considered percent differences greater than 4% for at least two time points as significant. The average error was 0.7% or less for corrected data of three replicates at each time point. Consequently, significant changes in the deuterium uptake rate were observed for PMF37 and its interacting sFcγRIIIa (Figs 3–5 and Supplementary Figs 3–5).

**Figure 6:**
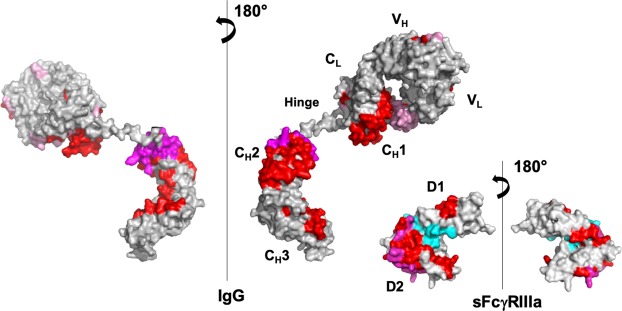
Mapping of HDX-MS data on 3D-structural models of IgG1 and sFcγRIIIa. We show the peptide segments exhibiting decreases in deuterium uptake rate upon interaction in red, magenta, or pink, and the peptide showing increased deuterium uptake in cyan, on a crystal structure of sFcγRIIIa extracted from Fc-sFcγRIIIa complex (PDB code:5XJE), and a homology model of PMF37 (shown as a half molecule composed of one light chain and one heavy chain). In the protected segments of PMF37, we classify residues into three types: the residues constituting the canonical sFcγRIIIa-binding sites in Fc (magenta), the remaining conserved residues among PMF37, trastuzumab, and rituximab (red), and the unconserved residues (pink). We classify the protected segments of sFcγRIIIa into two types; residues constituting the canonical Fc-binding sites (magenta) and the remaining conserved residues between FcγRIIIa and FcγRIIIb (red). The canonical binding sites are based on the crystal structure of Fc-sFcγRIIIa complex. We built the homology model based on crystal structures of the human anti-human immunodeficiency virus-1 gp120 IgG1 (PDB code: IHZH^54^) using SWISS-MODEL Workspace^55^. We prepared the molecular graphics using PyMOL (http://www.pymol.org/).

With regard to PMF37, we observed significant decreases in the deuterium uptake rate for Ser65-Leu72 in V_L_, Thr105-Glu124 and Thr163-Tyr177 in C_L_, and Phe54-Asn62 in V_H_, Pro127-Leu142, Asn159-Leu174, and Val186-Thr197 in C_H_1, Leu242-Leu251, Val263-Phe275, Tyr278-Thr300, and Tyr319-Glu333 in C_H_2, and Trp381-Leu398 in C_H_3. On the other hand, sFcγRIIIa showed a deuterium uptake rate reduction in Ile49-Tyr56 and Leu84-Leu92 in the membrane-distal D1 domain and Lys101-Leu118 and His119-Phe139 in the membrane-proximal D2 domain, whereas Phe11-Trp16 and Leu12-Asp23 in the D1 domain exhibited an increased uptake rate upon binding to PMF37. We map the HDX data on 3D-structural models of PMF37 and sFcγRIIIa (Fig. 6). Our results indicate protection against deuterium uptake observed not only in Fc but also in Fab, implying Fab’s involvement in interactions with sFcγRIIIa.

## Discussion

The interaction of FcγR with IgG is widely assumed to be mediated through the Fc region, as its name indicates. This concept was established from the 1960s through the 1970s, when FcγRs were putative molecules and their interactions with IgGs were characterized mainly by cell-based assays, such as rosette formation^8^. These studies demonstrated their interactions to be inhibited by the Fc fragments but not the Fab fragment, and were followed by molecular cloning of FcγRs and subsequently a number of structural studies using the Fc fragments in complex with the soluble forms of their cognate receptors^14,29^.

However, the earlier studies also pointed to the possible roles of Fab in modulating effector functions expressed by Fc^2^. For example, the Fc fragment isolated from human IgG4 was shown to have a greater ability to bind C1q than that of the intact IgG4, which was interpreted as steric obstruction of the C1q-binding site by the Fab arms^30^. Most notably, Birshtein et al. reported that the protein ICR16,—a mouse IgG variant having the C_H_1 domain of IgG2b and the remaining heavy chain constant region of IgG2a—did not inhibit rosette formation between a macrophage line and IgG2a-coated sheep red blood cells^31^. Based on this paradoxical observation, the Fab arms were thought to affect the functional capacity of the Fc region either by inducing conformational changes or by obscuring the putative FcγR-binding site in Fc. The present study unintentionally revisited this issue.

The HS-AFM data visualized the dwell times of IgG1 molecules on clustering FcγRIIIa to be significantly longer than those of their Fc fragments, indicating that their Fab portions stabilized the complexes formed with the receptor. Furthermore, the HDX-MS data detected a reduction in deuterium uptake not only in the canonical interaction sites, i.e., the hinge-proximal segments of the Fc C_H_2 domains and the protruding loops of the FcγRIIIa D2 domain^20–24^—but also in the Fab region, along with the FcγRIIIa D1 domain (Fig. 6). Based on these observations, we conclude that the Fab arm is directly involved in interactions with FcγRIIIa.

Sequence comparison of these putative FcγRIIIa-binding sites in the Fab region of IgG1 highlights the C_L_ and C_H_1 segments conserved among the three IgG1 antibodies—PMF37, trastuzumab, and rituximab—suggesting the secondary interaction is mediated most likely through the C_H_1 and C_L_ domains of IgG1 and the D1 domain of FcγRIIIa (Supplementary Fig. 1a,b). The additional IgG1-binding segment in the D1 domain of FcγRIIIa—that is, Ile49-Tyr56—is conserved in FcγRIIIb (Supplementary Fig. 1c), suggesting that the Fab-binding property is commonly shared by these FcγRIII receptors. However, contradictory HDX-MS data have been reported regarding sFcγRIIIa-induced microenvironmental change in the C_H_1 segments around the position 160 (Asn159-Leu174 in this study): Houde *et al*. reported that 157–164 (Val156-Leu163 in our numbering system) was protected upon sFcγRIIIa binding, consistent with our results, whereas, in contrast, Shi *et al*. did not shed light on such protection^32,33^. In these two reports, no significant difference in deuterium uptake has been described for the remaining parts in the Fab region. The apparent discrepancy among the results might be attributed to differences in HDX-MS experimental conditions, including protein concentrations, deuterium incubation time, pH, and temperature besides variations in glycoforms of the IgG1 and sFcγRIIIa samples. Intriguingly, Shi *et al*. showed that the C_H_1 segment Val150-Tyr162 (Val146-Trp158 in our numbering system) was exposed upon binding to sFcγRIIIa by a fast photochemical oxidation experiment, which enables sensitively probing fast dynamics in protein conformational change^33^. Their explanation of this result is that Fab and Fc regions are in close contact conformationally in its prebound state and Fab is released from the interaction with Fc upon binding to the receptor. A possible integrative interpretation of these data along with our data is that binding of the FcγRIIIa D2 domain to Fc causes transient exposure of the C_H_1 segment in either or both Fab arms, which is followed by association of one Fab arm with the D1 domain.

Our HDX-MS experiments also detected allosteric conformational changes in both IgG1 and sFcγRIIIa. We observed protection against deuterium uptake for the segments located at the interface between the C_H_2 and C_H_3 domains of IgG1-Fc—that is, Leu242-Leu251 and Trp381-Leu398, which are distal from the FcγRIIIa-binding sites. This is consistent with the structural data showing quaternary-structure deformation of Fc induced by binding to sFcγRIII^20–25,34^. Furthermore, we observed the enhanced solvent exposure for the Phe11-Trp16 and Leu12-Asp23 segments, which are located at the interface between the D1 and D2 domain of FcγRIIIa. This is also consistent with the crystal structures of sFcγRIIIb showing a domain rearrangement upon binding to IgG1-Fc^20,21,25,35^. We noted the putative FcγRIIIa-binding site in Fab to partially overlap with the area involved in interaction with protein G^36^, which has been reported to be dependent on antigen binding^37^. This suggests the intriguing possibility that antigen binding has potential impacts on the conformations of these sites, thereby allosterically affecting the Fab-FcγRIIIa interaction.

FcγRIIIa, expressed on NK cells, mediates ADCC, and is therefore considered to be a critical target of therapeutic antibodies for cancer treatments^3,38^. One of the promising approaches in attempting to improve therapeutic efficacy of IgG drugs is to enhance their affinities to FcγRIIIa. So far, such undertakings have been focused on the Fc portion of IgG by amino acid substitutions at the previously identified FcγRIIIa-binding site, and also by engineering of the N-glycans attached to Fc, best exemplified by the removal of the core fucose residue, causing ADCC enhancement^3,28,39–43^. Our findings in the present study offer a novel strategy for developing therapeutic IgG antibodies with higher affinities for FcγRIIIa by rational and evolutionary engineering targeting the previously unknown—but commonly shared—interaction sites in their Fab portions.

## Methods

### Materials

We purchased rituximab, an anti-CD20 mouse/human-chimeric IgG1 (G1m17,1; Km3)^44^, from Chugai Pharmaceutical Co., LTD. Trastuzumab, anti-HER2 humanized IgG1(G1m17,1; Km3)^45^, and PMF37, human anti-hepatitis A virus IgG1(G1m3; λ2)^46^, were expressed by the CHO-HcD6 and Baby Hamster Kidney (BHK) cell lines, respectively, according to previously described methods^46,47^. We cultivated the CHO-HcD6 cells in BalanCD^®^ CHO Growth A medium (Irvine Scientific) supplemented with 2 mM L-glutamine, 1% Penicillin-Streptomycin (Thermo fisher scientific), and 7.5 μg/ml puromycin (Nacalai tesque). We cultivated the BHK cells in OptiPRO™ SFM medium (Thermo fisher scientific) supplemented with 2 mM L-glutamine, 1% penicillin-streptomycin (Thermo fisher scientific), and 100 μg/ml hygromycin B (Wako). Following the growth of the cells, we applied the supernatant of medium to an nProtein A Sepharose Fast Flow column (GE Healthcare) and further purified IgG1 by gel filtration using a HiLoad 16/60 Superdex 200 pg column (GE Healthcare) with a 50 mM Tris-HCl, pH 8.0, buffer containing 150 mM NaCl.

For preparation of Fc fragments, we incubated IgG1 dissolved at a final concentration of 10 mg/ml in 75 mM phosphate buffer, pH 6.0, containing 75 mM NaCl, and 2 mM ethylenediaminetetraacetic acid, in the presence of papain (Merck) with an enzyme/substrate ratio of 2% at 37 °C for 12 h. We then terminated the reaction by adding 33 mM N-ehylmaleimide. We applied the reaction mixture to an nProtein A Sepharose Fast Flow (GE Healthcare) for isolation of Fc fragments. We further purified the Fc fragment by gel filtration using a Superdex 200 Increase 10/300 GL column (GE Healthcare). We checked purity of each of the Fc preparations by SDS-PAGE (Supplementary Fig. 6). For mock-treated controls, we incubated IgG1 under the same conditions except for the absence of papain.

According to the previous study^48^, we generated a construct of human sFcγRIIIa as recombinant glycoprotein with a C-terminal hexahistidine tag and two N-glycosylation sites at Asn45 and Asn162, while substituting the remaining three N-glycosylation sites—namely, Asn38, Asn74, and Asn169—with glutamine. Here, we refer to this recombinant sFcγRIIIa glycoprotein simply as sFcγRIIIa. We purchased the synthesized gene for sFcγRIIIa with an Igκ signal sequence from FASMAC and subcloned it into a pEHX1.2 vector (Toyobo), which we then used for protein production by dihydrofolate reductase (dhFr)-mediated gene amplification. We transfected the expression vector into the dhFr-deficient CHO cell line, CHO/dhFr- (ATCC® CRL-9096). After 48 h of transfection, we plated the transfected cells into 6-well plates for methotrexate (MTX) pressure selection. During the multi-round selection process, we gradually increased the MTX concentration up to 500 mM. We subjected the MTX-resistant cells to monoclonal screening by limited dilution to select clones with higher expression. We selected the high expression clones by ELISA using anti-His antibody (GE heltcare).

We cultured the high expression CHO cells in Dulbecco’s Modified Eagle’s medium (DMEM) with 10% fetal bovine serum and 500 mM MTX. After a 3-week cell culture, we applied the supernatant of the medium to a cOmplete His-Tag Purification Resin column (Roche) and further purified sFcγRIIIa by gel filtration using a HiLoad 16/60 Superdex 75 pg column with a 50 mM Tris-HCl, pH 8.0, buffer containing 150 mM NaCl. For desialylation, we incubated sFcγRIIIa in 50 mM sodium acetate (pH.5.5) and 150 mM NaCl at 37 °C for 12 h in the presence of one unit neuraminidase from *Arthrobacter ureafaciens* (Nacalai tesque) per 5 mg of sFcγRIIIa.

### HS-AFM observation and analysis

For the HS-AFM experiments, we used a laboratory-built high-speed atomic force microscope in tapping mode^49^ at room temperature. We used a small cantilever oscillating with a resonant frequency of ~0.6 MHz (in water), a spring constant of ~0.2 N m^−1^, and a quality factor of ~2 at the resonant frequency and thereby detected the variation of the oscillation amplitude by a two-element segmented photodiode. We fabricated an AFM tip on the cantilever using the electron beam deposition (EBD) method^50^. The length of the EBD tip was ~500 nm, and the tip apex radius was approximately 4 nm. We set the free oscillation amplitude of the cantilever at ~1 nm. We defined a set-point of amplitude for feedback control at approximately 90% of the free amplitude to prevent an unwanted disturbance of interactions of sFcγRIIIa with IgG1 or its Fc fragment. To immobilize sFcγRIIIa through the C-terminal hexahistidine tag, we treated the freshly cleaved mica surface with a droplet of 10 mM NiCl_2_. After treatment, we placed and incubated a droplet of sFcγRIIIa (approximately 2 μl) for five min; we then removed the sFcγRIIIa molecules unbound to the substrate by rinsing with a 50 mM Tris-HCl, pH 8.0, buffer containing 150 mM NaCl. After washing, we immersed the sample stage of HS-AFM in a chamber with approximately 70 μl of 50 mM Tris-HCl, pH 8.0, buffer containing 150 mM NaCl.

We measured the bound-state dwell time using successive HS-AFM images to estimate the binding time of IgG1 (or its Fc fragment) on sFcγRIIIa by monitoring their interactions as appearance or disappearance of bright spots in the HS-AFM images. We conducted all analysis using a custom software program based on IgorPro 6 (WaveMetrics, Inc., Lake Oswego, Ore., USA).

### HDX-MS analysis

As we performed previously^51,52^, optimal concentrations of proteins, estimated based on their dissociation constant, were employed for HDX-MS experiments. We performed HDX-MS analysis with an automated HDx3 system (LEAP Technologies) set up analogously to previously described protocol, with the syringe chiller on. We diluted protein solutions 20-fold with deuterated PBS (pD 8) and incubated them at 20 °C for various hydrogen/deuterium exchange time periods (namely, 30 sec, 60 sec, 180 sec, 600 sec, 3600 sec or 14400 sec). Here, we estimated the fraction of IgG1-sFcγRIIIa complex to be 95% or more under receptor-excess conditions [final concentrations: PMF37 (0.33 μM) and sFcγRIIIa (6.0 μM)] and 85% or more under IgG-excess conditions [final concentrations: PMF37 (3.7 μM) and sFcγRIIIa (2.4 μM)]. We quenched the exchange reaction at 1 °C by dropping the pH to 2.5 by mixing equal volume to diluted protein solution of 200 mM NaH_2_PO_4_ (Wako), 4 M GdnHCl (Wako), and 150 mM Tris (2-carboxyethyl) phosphine hydrochloride (Sigma Aldrich). We used the following columns and pump: Poroszyme Immobilized Pepsin Cartridge (2.1 × 30 mm) (Thermo Fisher Scientific); trap, Acclaim PepMap300 C18 5 mm (1 × 15 mm) (Thermo Fisher Scientific); analytical, Hypersil Gold (1 × 50 mm, 1.9 μm) (Thermo Fisher Scientific); and LC pump, Dionex Ultimate 3000 (Thermo Fisher Scientific). We set the loading pump (from the protease column to the trap column) at 50 μL/min with 0.1% aqueous formic acid. We set the gradient pump (from the trap column to the analytical column) from 10% to 25%, with 90% acetonitrile in 0.1% aqueous formic acid in 10 min at 45 μL/min. We washed all systems and lines each time between sample measurements with automated methods using 2 M GdnHCl with 100 mM citric acid, pH 2.3. We carried out mass spectrometric analyses using an Q Exactive HF-X (Thermo Fisher Scientific) with the capillary temperature at 275 °C, resolution 120,000 or 240,000 and mass range (m/z) 200–2000. We used Proteome Discoverer 2.2.0.387 (Thermo Fisher Scientific) for the peptide identification of non-deuterated samples prior to the HDX experiments. We used the HD-Examiner version 2.5.1 (Sierra Analytics) to extract centroid values from the MS raw data files for the HDX experiments. We processed and presented the data using Excel, and the results of three independent experiments were subjected to significant test according to the literature^53^.

## Supplementary information

Supplementary Video1

Supplementary Video2

Supplementary Video3

Supplementary Video4

Supplementary Video5

Supplementary Video6

Supplementary information

## References

[1] Dorrington KJ, Klein MH (1982). Binding sites for Fcγ receptors on immunoglobulin G and factors influencing their expression. Mol Immunol.

[2] Burton DR (1985). Immunoglobulin G: functional sites. Mol Immunol.

[3] Jefferis R, Lund J, Pound JD (1998). IgG-Fc-mediated effector functions: molecular definition of interaction sites for effector ligands and the role of glycosylation. Immunol Rev.

[4] Yang D, Kroe-Barrett R, Singh S, Roberts CJ, Laue TM (2017). IgG cooperativity - Is there allostery? Implications for antibody functions and therapeutic antibody development. MAbs.

[5] Jay Jacob, Bray Brinkley, Qi Yaozhi, Igbinigie Eseosaserea, Wu Hao, Li Jinping, Ren Gang (2018). IgG Antibody 3D Structures and Dynamics. Antibodies.

[6] Uhr JW (1965). Passive sensitization of lymphocytes and macrophages by antigen-antibody complexes. Proc Natl Acad Sci USA.

[7] Phillips-Quagliata JM, Levine BB, Uhr JW (1969). Studies on the mechanism of binding of immune complexes to phagocytes. Nature.

[8] Zuckerman SH, Douglas SD (1978). The characterization and functional significance of plasma membrane Fc Receptors. CRC Crit Rev Microbiol.

[9] Fridman WH (1992). Structural bases of Fcγ receptor functions. Immunol Rev.

[10] Hulett MD, Hogarth PM (1994). Molecular basis of Fc receptor function. Adv Immunol.

[11] Daeron M (1997). Fc receptor biology. Annu Rev Immunol.

[12] Bruhns P (2009). Specificity and affinity of human Fcγ receptors and their polymorphic variants for human IgG subclasses. Blood.

[13] Bruhns P (2012). FcR properties of mouse and human IgG receptors and their contribution to disease models. Blood.

[14] Hayes JM, Wormald MR, Rudd PM, Davey GP (2016). Fcγ receptors: glycobiology and therapeutic prospects. J Inflamm Res.

[15] Boros P (1991). IgM anti-FcγR autoantibodies trigger neutrophil degranulation. J Exp Med.

[16] Hundt M, Schmidt RE (1992). The glycosylphosphatidylinositol-linked Fcγ receptor III represents the dominant receptor structure for immune complex activation of neutrophils. Eur J Immunol.

[17] Galon J (1998). Identification of the cleavage site involved in production of plasma soluble Fcγ receptor type III (CD16). Eur J Immunol.

[18] Masuda M (2003). Measurement of soluble Fcγ receptor type IIIa derived from macrophages in plasma: increase in patients with rheumatoid arthritis. Clin Exp Immunol.

[19] Albanesi M, Daeron M (2012). The interactions of therapeutic antibodies with Fc receptors. Immunol Lett.

[20] Sondermann P, Huber R, Oosthuizen V, Jacob U (2000). The 3.2-A crystal structure of the human IgG1 Fc fragment-FcγRIII complex. Nature.

[21] Radaev S, Motyka S, Fridman WH, Sautes-Fridman C, Sun PD (2001). The structure of a human type III Fcγ receptor in complex with Fc. J Biol Chem.

[22] Mizushima T (2011). Structural basis for improved efficacy of therapeutic antibodies on defucosylation of their Fc glycans. Genes Cells.

[23] Ferrara C (2011). Unique carbohydrate-carbohydrate interactions are required for high affinity binding between FcγRIII and antibodies lacking core fucose. Proc Natl Acad Sci USA.

[24] Sakae Y (2017). Conformational effects of N-glycan core fucosylation of immunoglobulin G Fc region on its interaction with Fcγ receptor IIIa. Sci Rep.

[25] Roberts JT, Barb AW (2018). A single amino acid distorts the Fcγ receptor IIIb/CD16b structure upon binding immunoglobulin G1 and reduces affinity relative to CD16a. J Biol Chem.

[26] Niwa R (2004). Defucosylated chimeric anti-CC chemokine receptor 4 IgG1 with enhanced antibody-dependent cellular cytotoxicity shows potent therapeutic activity to T-cell leukemia and lymphoma. Cancer Res.

[27] Ferrara C, Stuart F, Sondermann P, Brunker P, Umana P (2006). The carbohydrate at FcγRIIIa Asn-162. An element required for high affinity binding to non-fucosylated IgG glycoforms. J Biol Chem.

[28] Yamane-Ohnuki N, Satoh M (2009). Production of therapeutic antibodies with controlled fucosylation. MAbs.

[29] Caaveiro JM, Kiyoshi M, Tsumoto K (2015). Structural analysis of Fc/FcγR complexes: a blueprint for antibody design. Immunol Rev.

[30] Isenman DE, Dorrington KJ, Painter RH (1975). The structure and function of immunoglobulin domains. II. The importance of interchain disulfide bonds and the possible role of molecular flexibility in the interaction between immunoglobulin G and complement. J Immunol.

[31] Birshtein BK, Campbell R, Diamond B (1982). Effects of immunoglobulin structure on Fc receptor binding: a mouse myeloma variant immunoglobulin with a gamma 2b-gamma 2a hybrid heavy chain having a complete gamma 2a Fc region fails to bind to gamma 2a Fc receptors on mouse macrophages. J Immunol.

[32] Houde D, Peng Y, Berkowitz SA, Engen JR (2010). Post-translational modifications differentially affect IgG1 conformation and receptor binding. Mol Cell Proteomics.

[33] Shi L, Liu T, Gross ML, Huang Y (2019). Recognition of Human IgG1 by Fcγ Receptors: Structural Insights from Hydrogen-Deuterium Exchange and Fast Photochemical Oxidation of Proteins Coupled with Mass Spectrometry. Biochemistry.

[34] Yogo R (2017). Characterization of conformational deformation-coupled interaction between immunoglobulin G1 Fc glycoprotein and a low-affinity Fcγ receptor by deuteration-assisted small-angle neutron scattering. Biochem Biophys Rep.

[35] Zhang Y (2000). Crystal structure of the extracellular domain of a human FcγRIII. Immunity.

[36] Derrick JP, Wigley DB (1994). The third IgG-binding domain from streptococcal protein G. An analysis by X-ray crystallography of the structure alone and in a complex with Fab. J Mol Biol.

[37] Oda M, Kozono H, Morii H, Azuma T (2003). Evidence of allosteric conformational changes in the antibody constant region upon antigen binding. Int Immunol.

[38] Wang W, Erbe AK, Hank JA, Morris ZS, Sondel PM (2015). NK Cell-Mediated Antibody-Dependent Cellular Cytotoxicity in Cancer Immunotherapy. Front Immunol.

[39] Shields RL (2002). Lack of fucose on human IgG1 N-linked oligosaccharide improves binding to human FcγRIII and antibody-dependent cellular toxicity. J Biol Chem.

[40] Shinkawa T (2003). The absence of fucose but not the presence of galactose or bisecting N-acetylglucosamine of human IgG1 complex-type oligosaccharides shows the critical role of enhancing antibody-dependent cellular cytotoxicity. J Biol Chem.

[41] Richards JO (2008). Optimization of antibody binding to FcgammaRIIa enhances macrophage phagocytosis of tumor cells. Mol Cancer Ther.

[42] Mimoto F (2013). Novel asymmetrically engineered antibody Fc variant with superior FcγR binding affinity and specificity compared with afucosylated Fc variant. MAbs.

[43] Isoda Y (2015). Importance of the Side Chain at Position 296 of Antibody Fc in Interactions with FcγRIIIa and Other Fcγ Receptors. PLoS One.

[44] McLaughlin P (1998). Rituximab chimeric anti-CD20 monoclonal antibody therapy for relapsed indolent lymphoma: half of patients respond to a four-dose treatment program. J Clin Oncol.

[45] Molina MA (2001). Trastuzumab (herceptin), a humanized anti-Her2 receptor monoclonal antibody, inhibits basal and activated Her2 ectodomain cleavage in breast cancer cells. Cancer Res.

[46] Lewis AP (1993). Rescue, expression, and analysis of a neutralizing human anti-hepatitis A virus monoclonal antibody. J Immunol.

[47] Onitsuka M, Omasa T (2015). Rapid evaluation of N-glycosylation status of antibodies with chemiluminescent lectin-binding assay. J Biosci Bioeng.

[48] Shibata-Koyama M (2009). The N-linked oligosaccharide at Fc gamma RIIIa Asn-45: an inhibitory element for high FcγRIIIa binding affinity to IgG glycoforms lacking core fucosylation. Glycobiology.

[49] Uchihashi T, Kodera N, Ando T (2012). Guide to video recording of structure dynamics and dynamic processes of proteins by high-speed atomic force microscopy. Nat Protoc.

[50] Koops HWP, Weiel R, Kern DP, Baum TH (1988). High-Resolution Electron-Beam Induced Deposition. Journal of Vacuum Science & Technology B: Microelectronics Processing and Phenomena.

[51] Uchiyama S (2015). Structural Basis for Dimer Formation of Human Condensin Structural Maintenance of Chromosome Proteins and Its Implications for Single-stranded DNA Recognition. J Biol Chem.

[52] Fujikawa A (2016). Small-molecule inhibition of PTPRZ reduces tumor growth in a rat model of glioblastoma. Sci Rep.

[53] Houde D, Berkowitz SA, Engen JR (2011). The Utility of Hydrogen/Deuterium Exchange Mass Spectrometry in Biopharmaceutical Comparability Studies. J. Pharm. Sci..

[54] Saphire EO (2001). Crystal structure of a neutralizing human IgG against HIV-1: a template for vaccine design. Science.

[55] Waterhouse A (2018). SWISS-MODEL: homology modelling of protein structures and complexes. Nucleic Acids Res.

