# Supplementary material for: The Fab portion of immunoglobulin G contributes to its binding to Fcγ receptor III
Source: Sci Rep. 2019 Aug 16;9:11957. doi: 10.1038/s41598-019-48323-w (PMC6697678; doi:10.1038/s41598-019-48323-w)
Supplement: Supplementary file 7 — Supplementary information [file 41598_2019_48323_MOESM7_ESM.pdf]

Supplementary information

The Fab portion of immunoglobulin G contributes to its binding to Fcγ receptor III

Rina Yogo [1, 2], Yuki Yamaguchi [3], Hiroki Watanabe [1], Hirokazu Yagi [2], Tadashi Satoh [2], Mahito Nakanishi [4], Masayoshi Onitsuka [5], Takeshi Omasa [3], Mari Shimada [3], Takahiro Maruno [3], Tetsuo Torisu [3], Shio Watanabe [6], Daisuke Higo [6], Takayuki Uchihashi [1,7], Saeko Yanaka [1, 2], Susumu Uchiyama [1, 3], and Koichi Kato [1, 2]

[1] Exploratory Research Center on Life and Living Systems (ExCELLS), National Institutes of Natural Sciences, 5-1 Higashiyama, Myodaiji, Okazaki, 444-8787, Japan; yogo@ims.ac.jp, hwatanabe@ims.ac.jp, saeko-yanaka@ims.ac.jp, kkatonmr@ims.ac.jp

[2] Graduate School of Pharmaceutical Sciences, Nagoya City University, 3-1 Tanabe-dori, Mizuho-ku, Nagoya, Aichi, 467-8603, Japan; hyagi@phar.nagoya-cu.ac.jp, tadashisatoh@phar.nagoya-cu.ac.jp

[3] Graduate School of Engineering, Osaka University, 2-1 Yamadaoka, Suita, Osaka, 565-0871, Japan; yuki.yamaguchi@bio.eng.osaka-u.ac.jp, omasa@bio.eng.osaka-u.ac.jp, marihorn211@gmail.com, tmaruno@bio.eng.osaka-u.ac.jp, tetsuo.torisu@bio.eng.osaka-u.ac.jp, suchi@bio.eng.osaka-u.ac.jp

[4] Biotechnology Research Institute for Drug Discovery, National Institute of Advanced Industrial Science and Technology (AIST), 1-1-1 Higashi, Central 5, Tsukuba, Ibaraki, 305-8565, Japan; mahito-nakanishi@aist.go.jp

[5] Graduate School of Technology, Industrial and Social Sciences, Tokushima University, Minamijosanjima-cho 2-1, Tokushima, 770-8513, Japan. onitsuka@tokushima-u.ac.jp.

[6] Thermo Fisher Scientific, 3-9 Moriya-cho, Kanagawa-ku, Yokohama-shi, Kanagawa, 221-0022, Japan; daisuke.higo@thermofisher.com, shio.watanabe@thermofisher.com

[7] Department of Physics, Nagoya University, Chikusa-ku, Nagoya, Aichi, 464-8602, Japan; uchihast@d.phys.nagoya-u.ac.jp

Supplementary Figures

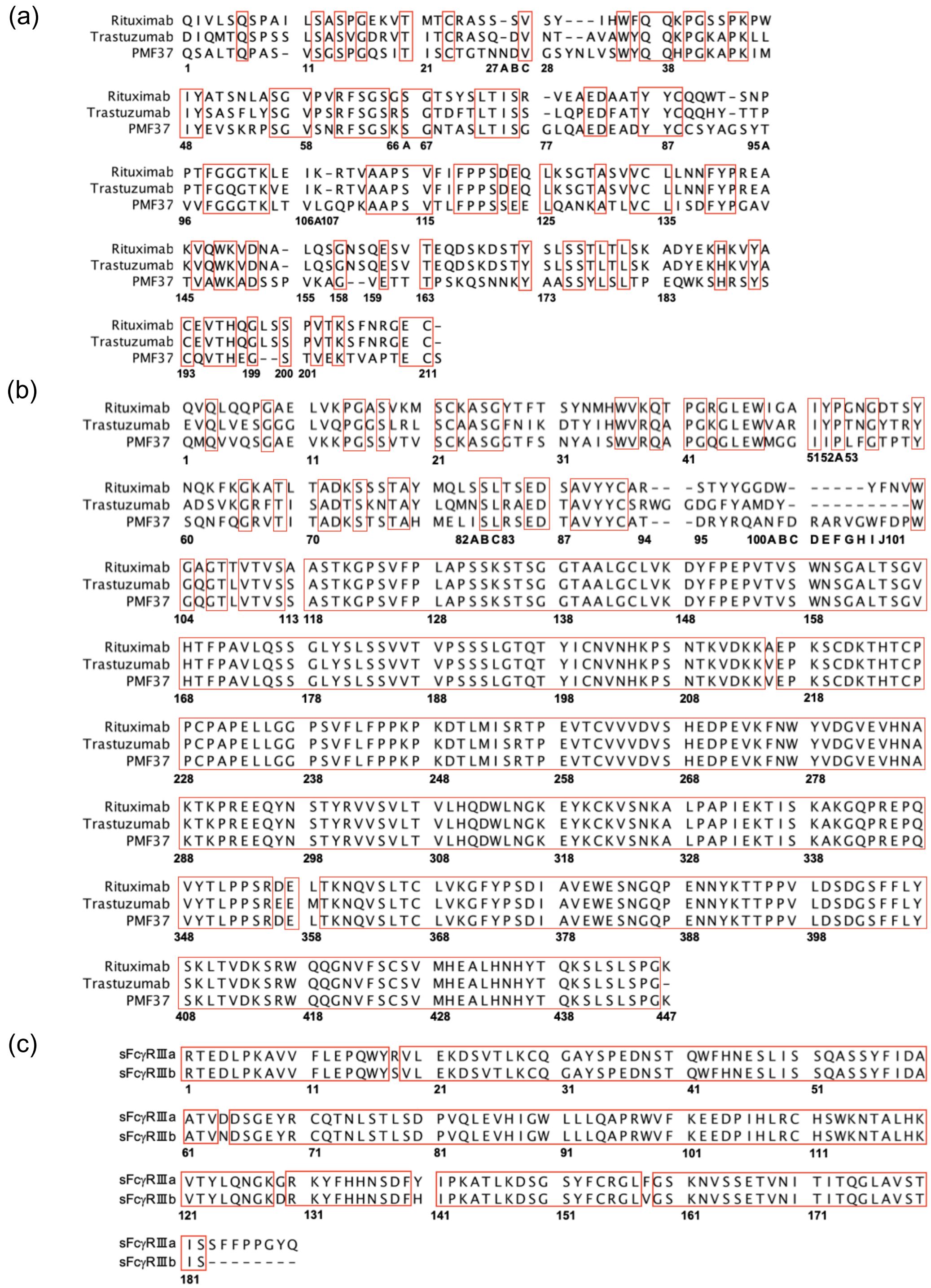

Supplementary Figure 1

Sequence alignments of (a) the light chains and (b) the heavy chains of rituximab, trastuzumab, and PMF37 and (c) sFcγRIIIa and sFcγRIIIb. The numbering system used in the present study for the constant region is based on human myeloma protein Eu [1]. The convention of Kabat et al. (1987) has been followed for the numbering of the variable regions [2]. Conserved residues are boxed in red.

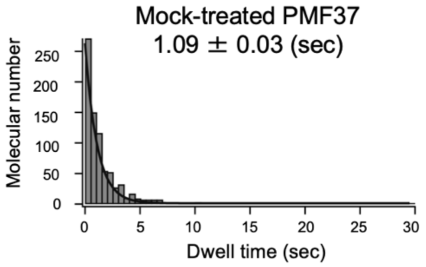

Supplementary Figure 2

Dwell time of mock-treated PMF37 on sFcγRIIIa.

(a)

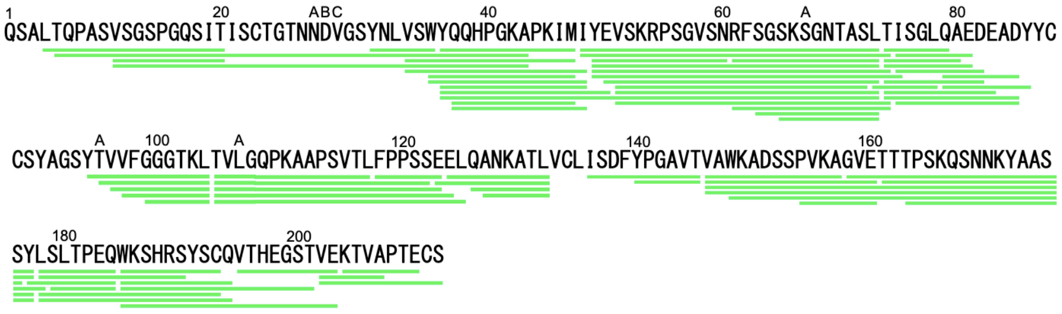
1
20
ABC
40
60
A
80
QSALTQPASVSGSPGQSITISCTGTNNDVGSYNLVSWYQQHPGKAPKIMIYEVSKRPSGVSNRFSGSKSGNTASLTISGLQAEDEADYYC
A
100
A
120
140
160
CSYAGSYTVVFGGGTKLTVLGQPKAAPSVTLFPPSSEELQANKATLVCLISDFYPGAVTVAWKADSSPVKAGVETTTPSKQSNNKYAAS
180
200
SYLSLTPEQWKSHRSYSCQVTHEGSTVEKTVAPTECS

(b)

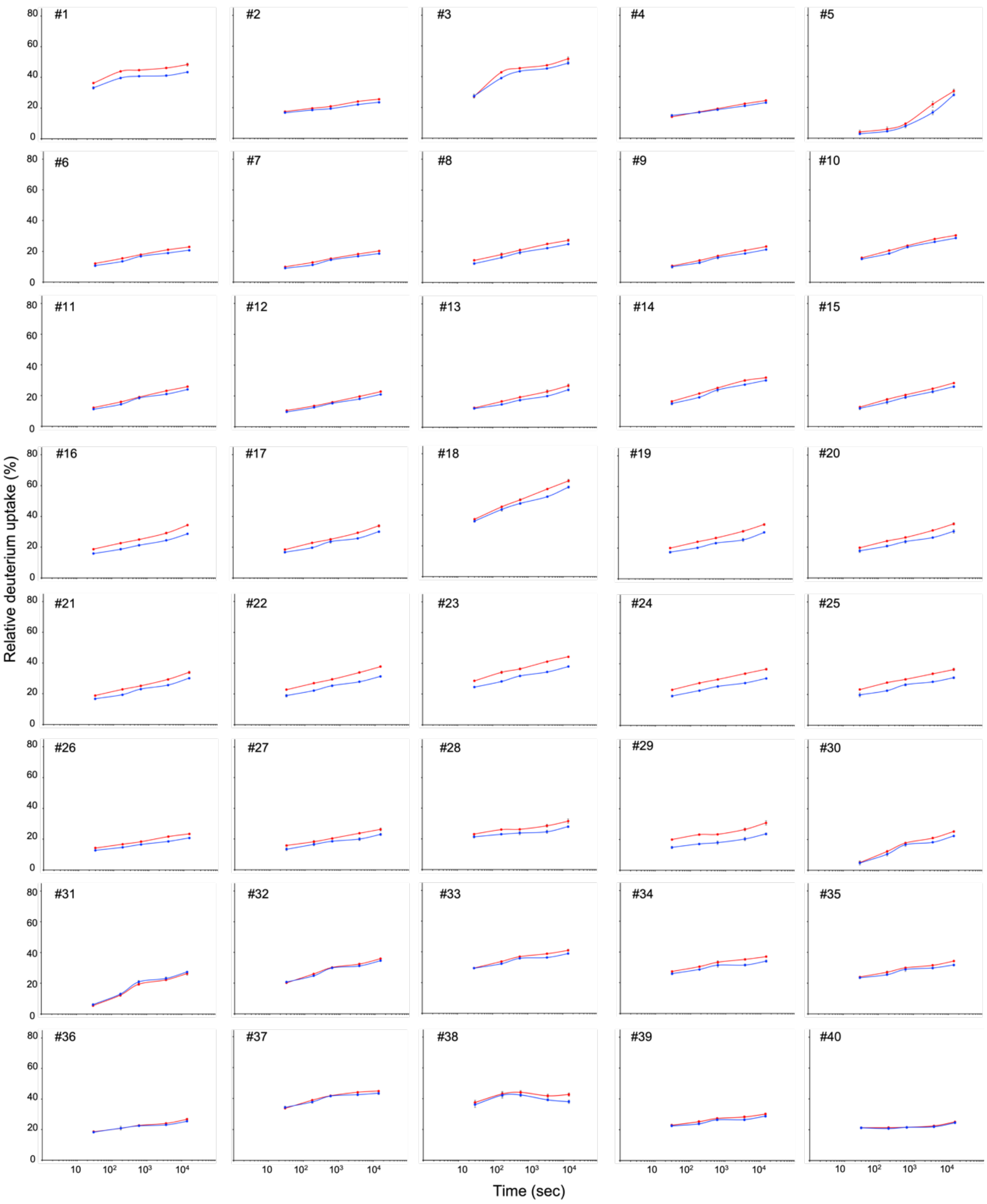
#1
#2
#3
#4
#5
#6
#7
#8
#9
#10
#11
#12
#13
#14
#15
#16
#17
#18
#19
#20
#21
#22
#23
#24
#25
#26
#27
#28
#29
#30
#31
#32
#33
#34
#35
#36
#37
#38
#39
#40
0
20
40
60
80
10
10²
10³
10⁴
Relative deuterium uptake (%)
Time (sec)

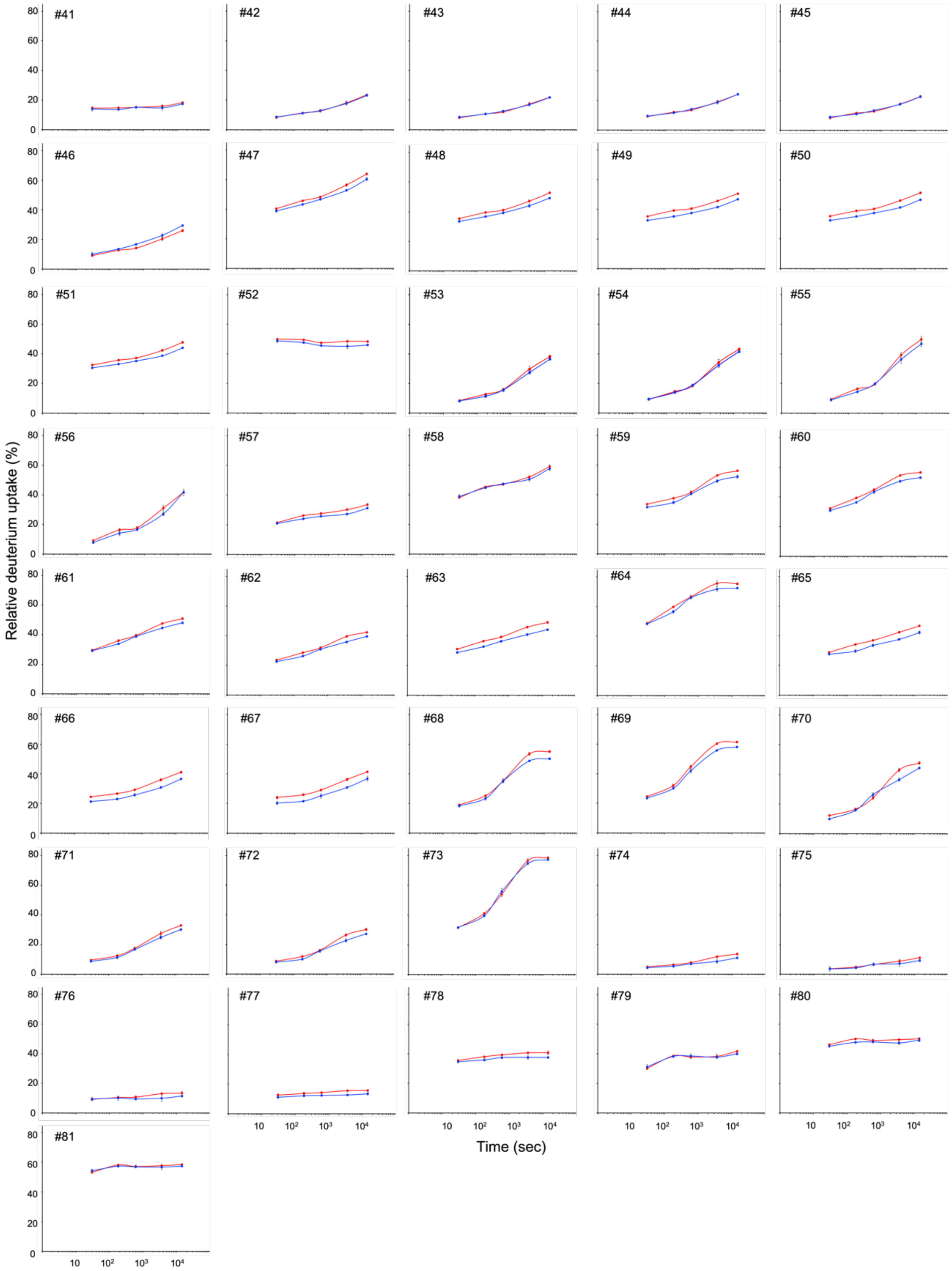

Supplementary Figure 3

HDX-MS data of the light chain. (a) Coverage map of identified peptides and (b) time-dependent deuterium uptake graphs of all peptides used for deuterium uptake comparison between free (red) and complexed states (blue).

(a)

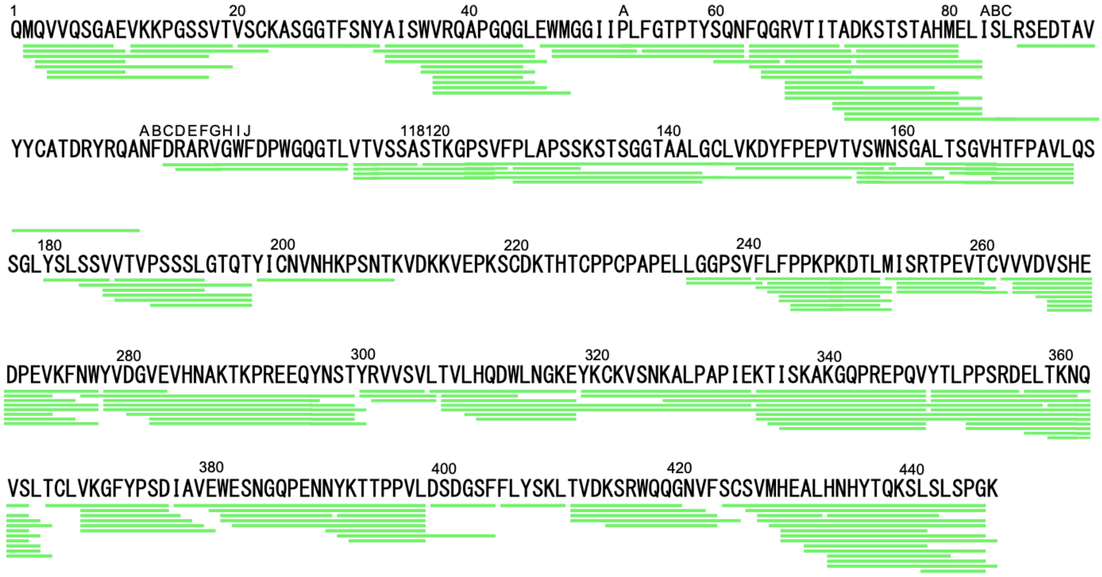

1 20 40 A 60 80 ABC
QMQVVQSGAEVKKPGSSVTVSCKASGGTFSNYAISWVRQAPGQGLEWMGGIIPLFGTPTYSQNFQGRVTITADKSTSTAHMELISLRSEDTAV
ABCDEFGHIJ 118120 140 160
YYCATDRYRQANFDRARVGWFDPWGQGTLVTVSSASTKGPSVFPLAPSSKSTSGGTAALGCLVKDYFPEPVTVSWNSGALTSGVHTFPAVLQS
180 200 220 240 260
SGLYSLSSVVTVPSSSLGTQTYICNVNHKPSNTKVDKKVEPKSCDKTHTCPPCPAPELLGGPSVFLFPPKPKDTLMISRTPEVTCVVVDVSHE
280 300 320 340 360
DPEVKFNWYVDGVEVHNAKTKPREEQYNSTYRVVSVLTVLHQDWLNGKEYKCKVSNKALPAPIEKTISKAKGQPREPQVYTLPPSRDELTKNQ
380 400 420 440
VSLTCLVKGFYPSDIAVEWESNGQPENNYKTTPPVLDSDGSFFLYSKLTVDKSRWQQGNVFSCSVMHEALHNHYTQKSLSLSPGK

(b)

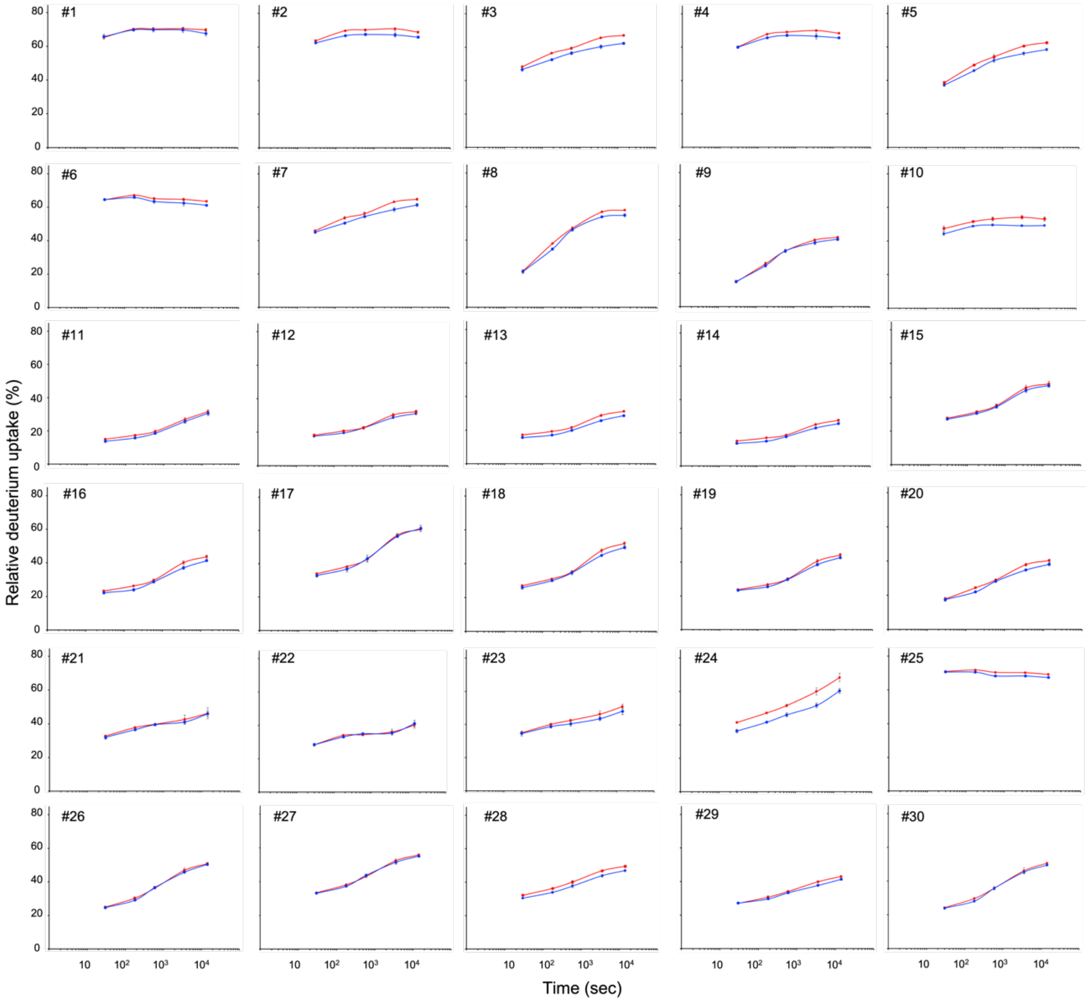

#1 #2 #3 #4 #5 #6 #7 #8 #9 #10 #11 #12 #13 #14 #15 #16 #17 #18 #19 #20 #21 #22 #23 #24 #25 #26 #27 #28 #29 #30
0 20 40 60 80
Relative deuterium uptake (%)
10 10² 10³ 10⁴
Time (sec)

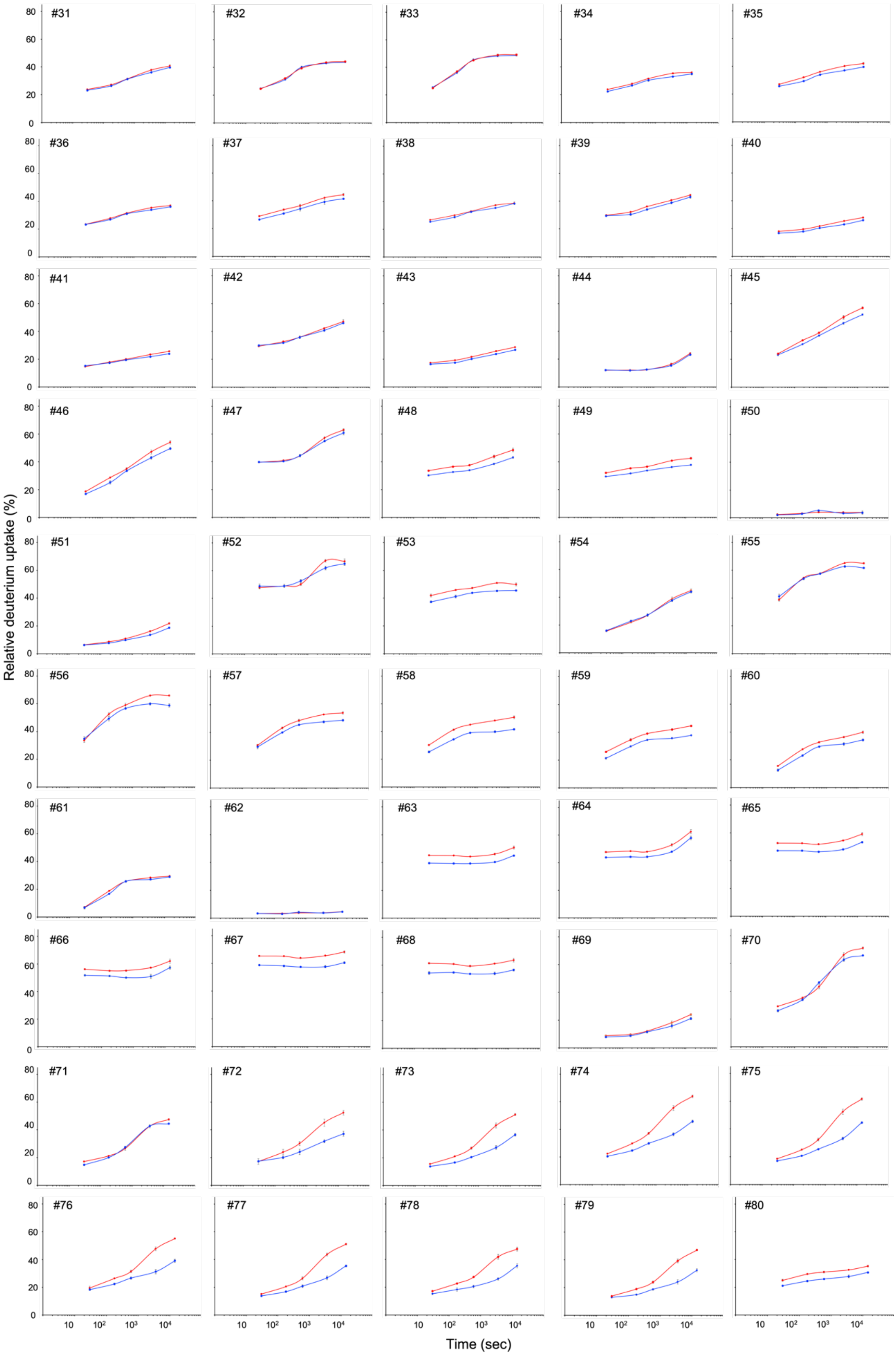

#31
#32
#33
#34
#35
#36
#37
#38
#39
#40
#41
#42
#43
#44
#45
#46
#47
#48
#49
#50
#51
#52
#53
#54
#55
#56
#57
#58
#59
#60
#61
#62
#63
#64
#65
#66
#67
#68
#69
#70
#71
#72
#73
#74
#75
#76
#77
#78
#79
#80
Relative deuterium uptake (%)
Time (sec)
0
20
40
60
80
10
10^2
10^3
10^4

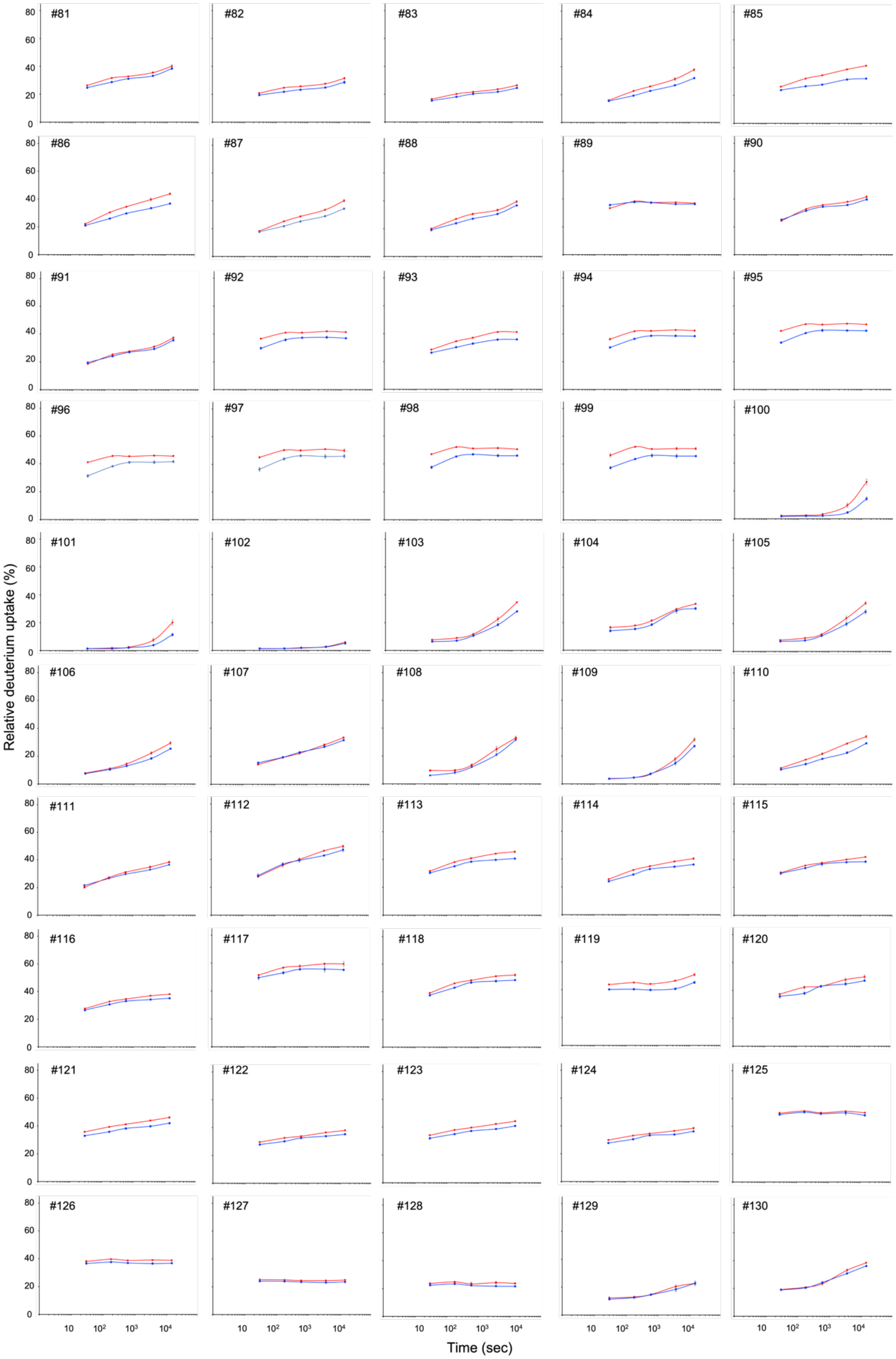

#81
#82
#83
#84
#85
#86
#87
#88
#89
#90
#91
#92
#93
#94
#95
#96
#97
#98
#99
#100
#101
#102
#103
#104
#105
#106
#107
#108
#109
#110
#111
#112
#113
#114
#115
#116
#117
#118
#119
#120
#121
#122
#123
#124
#125
#126
#127
#128
#129
#130
Relative deuterium uptake (%)
Time (sec)

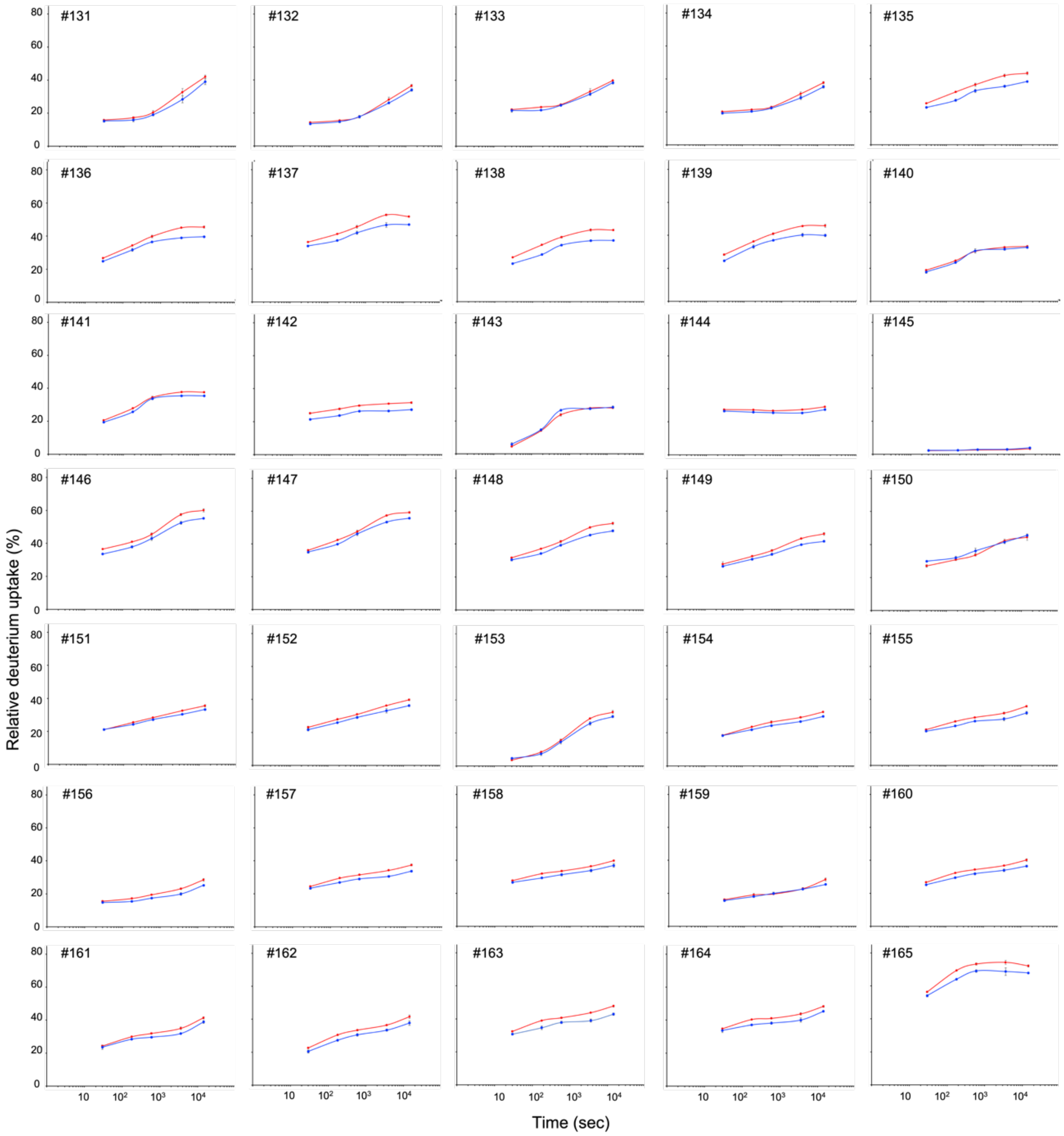

Supplementary Figure 4

HDX-MS data of the heavy chain. (a) Coverage map of identified peptides and (b) time-dependent deuterium uptake graphs of all peptides used for deuterium uptake comparison between free (red) and complexed states (blue).

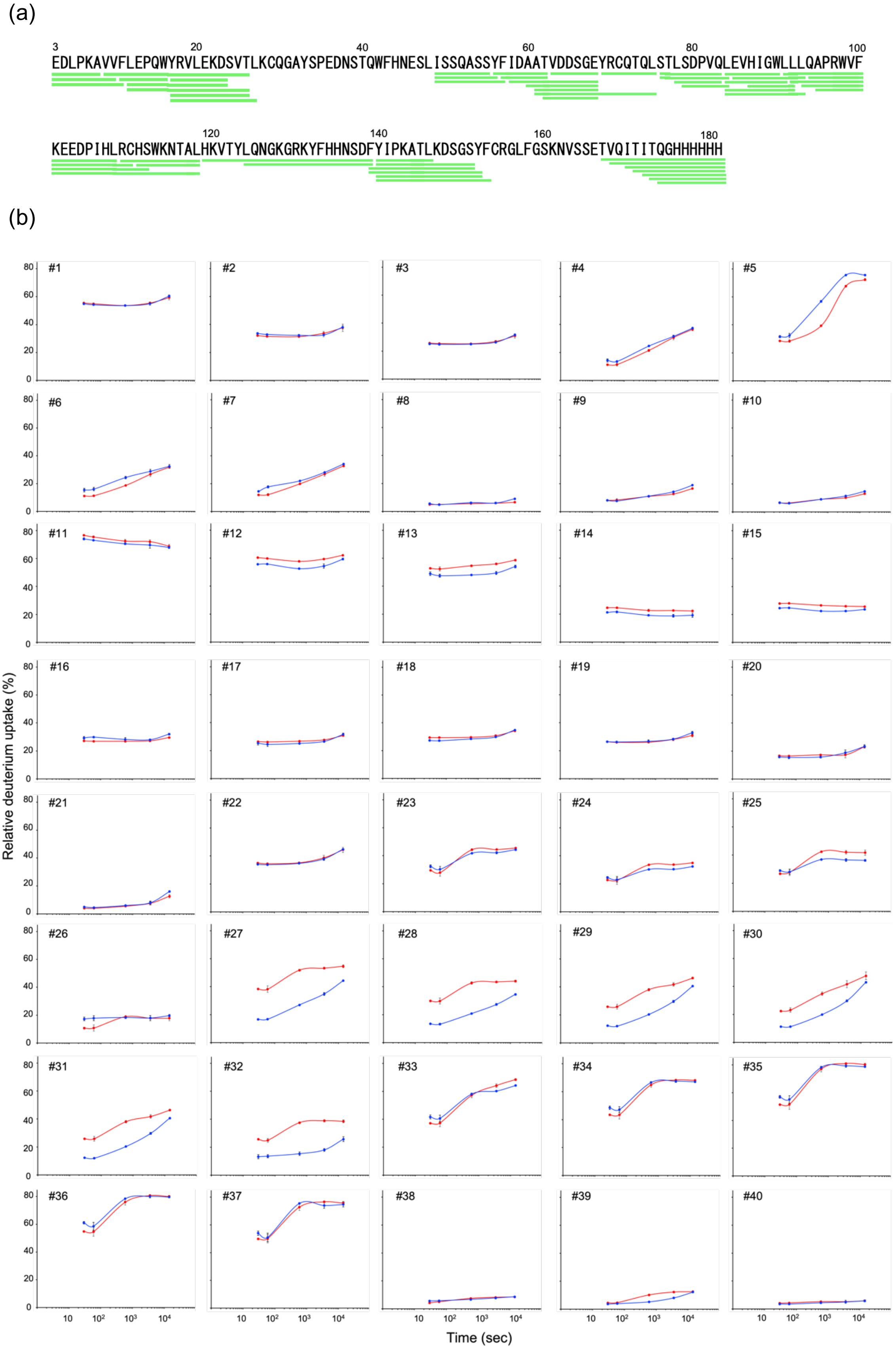

(a)
3
20
40
60
80
100
EDLPKAVVFLEPQWYRVLEKDSVTLKCQGAYSPEDNSTQWFHNESLISSQASSYFIDAATVDDSGEYRCQTQLSTLSDPVQLEVHIGWLLLQAPRWVF
120
140
160
180
KEEDPIHLRCHSWKNTALHKVTYLQNGKGRKYFHHNSDFYIPKATLKDSGSYFCRGLFGSKNVSSETVQITITQGHHHHHH
(b)
Relative deuterium uptake (%)
Time (sec)
#1 #2 #3 #4 #5 #6 #7 #8 #9 #10 #11 #12 #13 #14 #15 #16 #17 #18 #19 #20 #21 #22 #23 #24 #25 #26 #27 #28 #29 #30 #31 #32 #33 #34 #35 #36 #37 #38 #39 #40
0 20 40 60 80
10 10^2 10^3 10^4

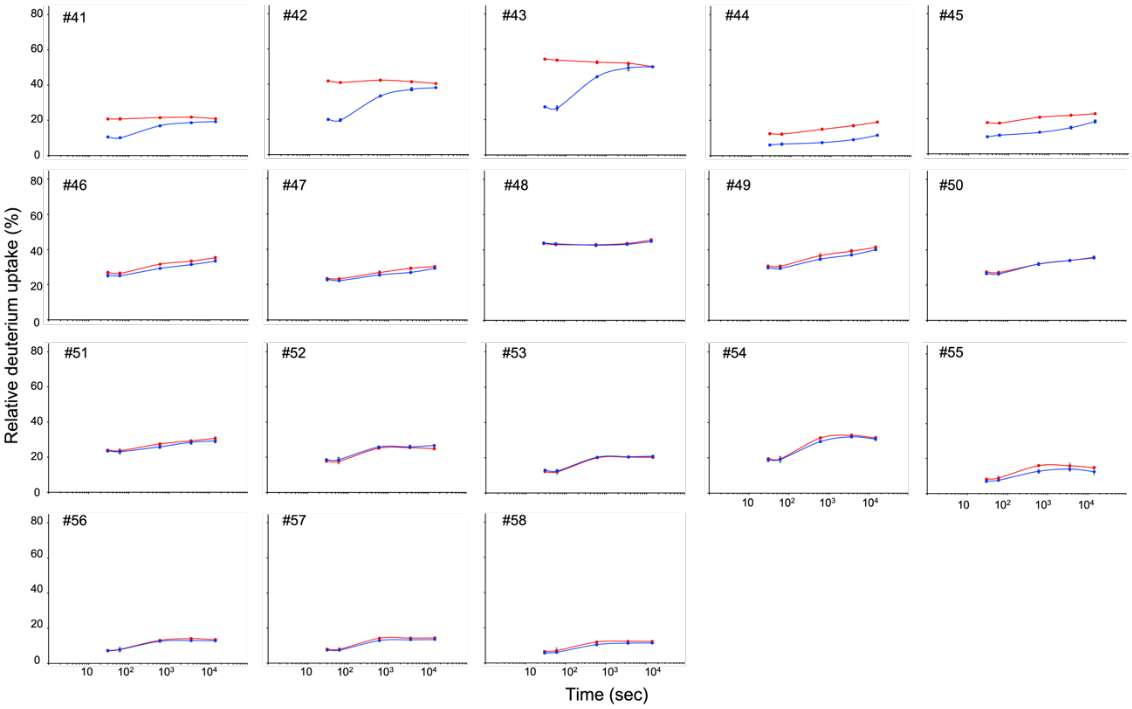

Supplementary Figure 5

HDX-MS data of sFcγRIIIa. (a) Coverage map of identified peptides and (b) time-dependent deuterium uptake graphs of all peptides used for deuterium uptake comparison between free (red) and complexed states (blue).

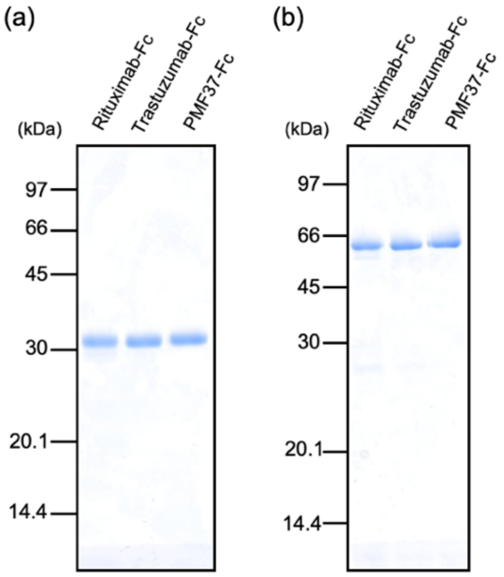

Supplementary Figure 6

SDS-PAGE of the Fc preparations under (a) reducing and (b) non-reducing conditions.

(a)

Peptide number	Sequence	Peptide number	Sequence	Peptide number	Sequence	Peptide number	Sequence
#1	L4-T20	#23	V51-S71	#45	V97-L104	#67	T163-Y177
#2	T5-A43	#24	V51-L72	#46	G99-L104	#68	Y177-Q184
#3	V11-T20	#25	V51-T73	#47	T105-L117	#69	L178-Q184
#4	V11-A43	#26	R61-L72	#48	T105-S122	#70	L178-S190
#5	Y30-W35	#27	R61-T73	#49	T105-E123	#71	L178-C193
#6	V33-M47	#28	S63-L72	#50	T105-E124	#72	L178-Q194
#7	V33-I48	#29	S65-L72	#51	T105-L125	#73	S179-Q184
#8	W35-M47	#30	L72-L78	#52	F118-E123	#74	W185-C193
#9	W35-I48	#31	T73-Q79	#53	E123-L132	#75	W185-Q194
#10	Y36-M47	#32	T73-A80	#54	E124-L132	#76	W185-T201
#11	Y36-I48	#33	T73-E81	#55	Q126-L132	#77	W185-E203
#12	Y36-E50	#34	T73-D82	#56	A127-L132	#78	V195-E203
#13	Y36-L72	#35	T73-E83	#57	I136-T145	#79	V202-A207
#14	Q37-M47	#36	T73-D85	#58	Y140-T145	#80	V202-S212
#15	Q37-I48	#37	I74-E81	#59	V146-A157	#81	K204-E210
#16	I48-L72	#38	I74-D82	#60	V146-E160		
#17	I48-T73	#39	I74-D85	#61	V146-S176		
#18	Y49-N60	#40	Q79-D85	#62	V146-L178		
#19	Y49-L72	#41	Q79-Y86	#63	W148-Y177		
#20	Y49-T73	#42	Y95-L104	#64	P154-E160		
#21	Y49-I74	#43	T95$_{A}$-L104	#65	G158-Y177		
#22	E50-L72	#44	V96-L104	#66	T161-Y177		

(b)

Peptide number	Sequence	Peptide number	Sequence	Peptide number	Sequence	Peptide number	Sequence	Peptide number	Sequence
#1	M2-A9	#35	R66-M80	#69	Y198-T209	#103	L306-E318	#137	W381-N390
#2	M2-E10	#36	R66-L82	#70	L235-V240	#104	T307-W313	#138	W381-L398
#3	M2-S17	#37	T70-M80	#71	L235-F241	#105	T307-E318	#139	E382-L398
#4	Q3-E10	#38	T70-L82	#72	F241-L251	#106	T307-E333	#140	N389-L398
#5	Q3-T19	#39	A71-M80	#73	F241-M252	#107	T307-V348	#141	Y391-L398
#6	V4-E10	#40	A71-L82	#74	L242-L251	#108	L309-E318	#142	Y391-F404
#7	V4-S17	#41	A71-A100	#75	L242-M252	#109	H310-E318	#143	K392-L398
#8	V11-T19	#42	D72-M80	#76	F243-L251	#110	Y319-E333	#144	D399-F404
#9	V11-C22	#43	D72-L82	#77	F243-M252	#111	Y319-V348	#145	F405-L410
#10	V20-Y32	#44	R83-V89	#78	P244-L251	#112	K326-V348	#146	T411-G420
#11	Y32-L45	#45	D100$_{C}$-L108	#79	P244-M252	#113	K334-V348	#147	T411-V422
#12	A33-G44	#46	R100$_{D}$-L108	#80	M252-C261	#114	K334-L358	#148	T411-F423
#13	A33-L45	#47	V109-A118	#81	I253-T260	#115	K334-S364	#149	T411-C425
#14	A33-E46	#48	V109-F126	#82	I253-C261	#116	K334-L365	#150	K414-F423
#15	W36-G44	#49	V109-L142	#83	I253-V262	#117	T335-V348	#151	S424-G446
#16	W36-L45	#50	V109-T155	#84	V262-W277	#118	I336-V348	#152	S426-G446
#17	V37-G44	#51	S119-N159	#85	V263-V273	#119	Y349-D356	#153	V427-L432
#18	V37-L45	#52	P127-S132	#86	V263-F275	#120	Y349-N361	#154	V427-G446
#19	V37-E46	#53	P127-L142	#87	V263-W277	#121	Y349-S364	#155	M428-G446
#20	V37-M48	#54	V146-W158	#88	D265-W277	#122	Y349-T366	#156	H429-L441
#21	E46-N62	#55	V156-A162	#89	V266-V273	#123	P352-S364	#157	H429-G446
#22	W47-L53	#56	V156-L163	#90	V266-F275	#124	P352-L365	#158	H429-K447
#23	W47-N62	#57	V156-L174	#91	V266-W277	#125	E357-S364	#159	A431-L443
#24	F54-N62	#58	N159-L174	#92	N276-T299	#126	E357-L365	#160	A431-G446
#25	S60-G65	#59	A162-L174	#93	Y278-E283	#127	T359-L365	#161	H433-S444
#26	F63-T70	#60	T164-L174	#94	Y278-Y296	#128	T359-T366	#162	H433-L443
#27	F63-A71	#61	H168-L174	#95	Y278-T299	#129	T366-D376	#163	H433-G446
#28	F63-M80	#62	Y180-V185	#96	Y278-Y300	#130	V369-D376	#164	H433-K447
#29	F63-L82	#63	S183-T197	#97	D280-T299	#131	V369-I377	#165	L441-G446
#30	Q64-A71	#64	V185-L193	#98	V282-T299	#132	V369-A378		
#31	Q64-L82	#65	V185-T197	#99	V282-Y300	#133	V369-V379		
#32	R66-A71	#66	V186-L193	#100	Y300-V305	#134	V369-E380		
#33	R66-D72	#67	V186-T197	#101	Y300-L306	#135	I377-L398		
#34	R66-A78	#68	P189-T197	#102	R301-L306	#136	E382-L398		

(c)

Peptide number	Sequence	Peptide number	Sequence	Peptide number	Sequence
#1	E3-A8	#23	S76-Q83	#45	L124-F139
#2	E3-V10	#24	S76-L84	#46	F139-S151
#3	E3-F11	#25	L78-Q83	#47	F139-Y152
#4	V9-T26	#26	S79-L84	#48	Y140-L146
#5	F11-W16	#27	L84-W90	#49	Y140-S151
#6	L12-D23	#28	L84-L91	#50	Y140-Y152
#7	L12-T26	#29	L84-L92	#51	Y140-F153
#8	Y17-D23	#30	L84-L93	#52	T167-H181
#9	Y17-T26	#31	E85-L93	#53	V168-H181
#10	Y17-L27	#32	H87-L92	#54	I170-H181
#11	I49-S55	#33	L91-F100	#55	T171-H181
#12	I49-Y56	#34	L92-F100	#56	I172-H181
#13	I49-F57	#35	L93-F100	#57	T173-H181
#14	Y56-T62	#36	Q94-F100	#58	Q174-H181
#15	F57-T62	#37	A95-F100		
#16	I58-E68	#38	K101-L108		
#17	A60-E68	#39	K101-C110		
#18	A61-E68	#40	K101-S112		
#19	A61-L75	#41	K101-L118		
#20	T62-E68	#42	R109-L118		
#21	V63-E68	#43	H111-L118		
#22	Y69-L75	#44	H119-F139		

Supplementary Table

Peptide identifications in (a) the light chain and (b) the heavy chain of PMF37 and (c) sFcγRIIIa.

Supplementary References

1. Edelman GM, Cunningham BA, Gall WE, Gottlieb PD, Rutishauser U, Waxdal MJ. The covalent structure of an entire gammaG immunoglobulin molecule. *Proc Natl Acad Sci U S A* **63**, 78-85 (1969).

2. Rabat, E. A., Wu, T. T., Reid-Miller, M., Perry, . M., & Gottesman, K. S. *Sequences of Proteins of Immu- nological Interest*, 4th ed., *U.S. Department of Health and Human Services, National Institutes of Health, Washington, DC.* (1987)
